# Supplementary figures and images for: High-quality reference genome of Fasciola gigantica: Insights into the genomic signatures of transposon-mediated evolution and specific parasitic adaption in tropical regions
Source: PLoS Negl Trop Dis. 2021 Oct 5;15(10):e0009750. doi: 10.1371/journal.pntd.0009750 (PMC8519440; doi:10.1371/journal.pntd.0009750)

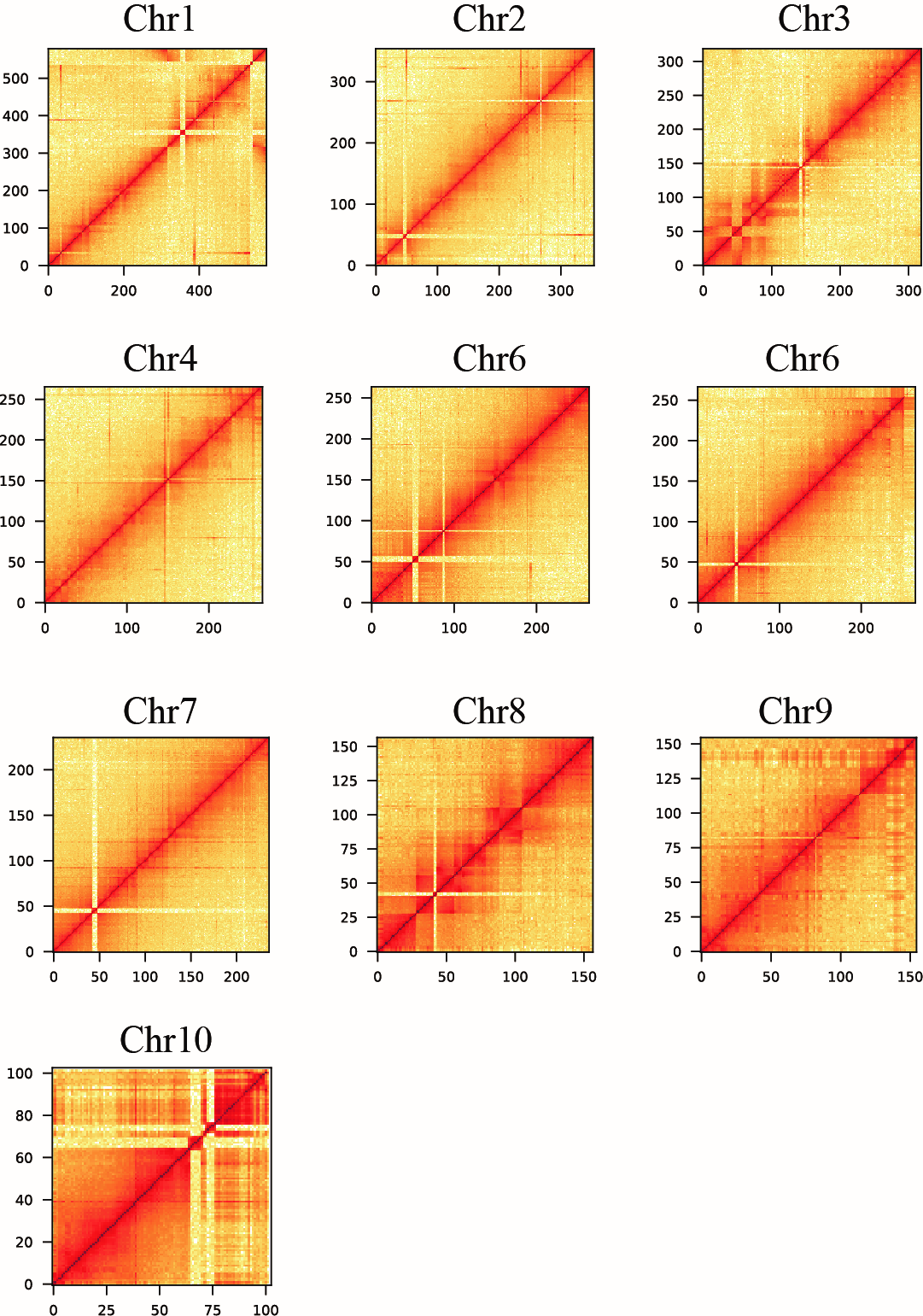

Supplement: S1 Fig — (TIF) [file pntd.0009750.s001.tif]

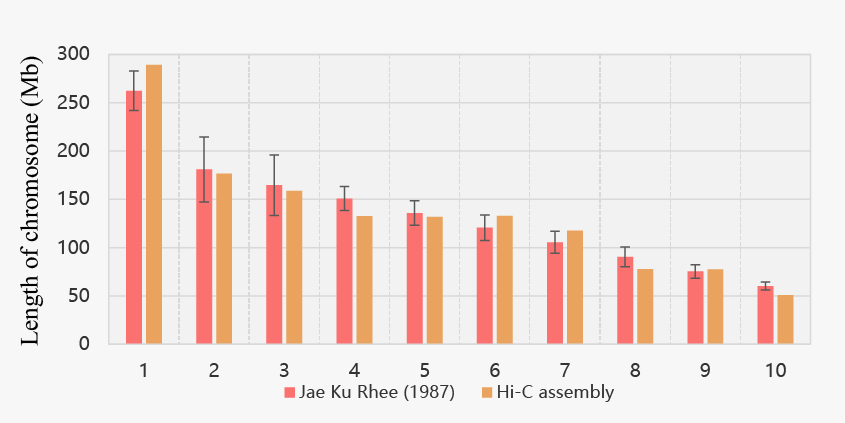

Supplement: S2 Fig — (TIF) [file pntd.0009750.s002.tif]

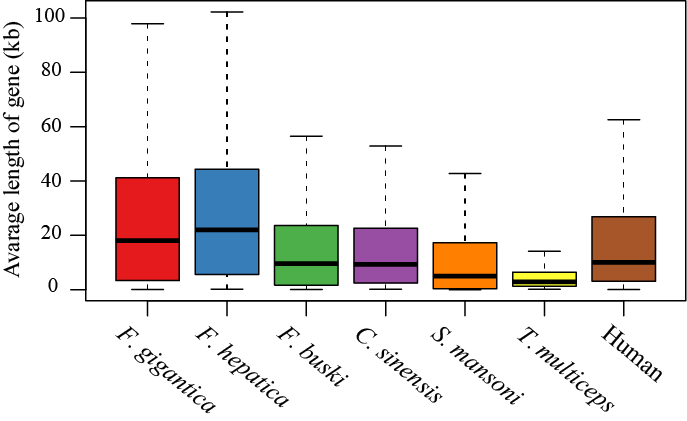

Supplement: S3 Fig — (TIF) [file pntd.0009750.s003.tif]

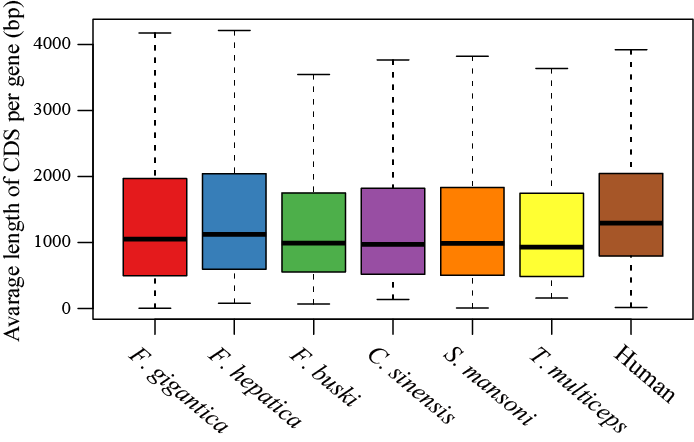

Supplement: S4 Fig — (TIF) [file pntd.0009750.s004.tif]

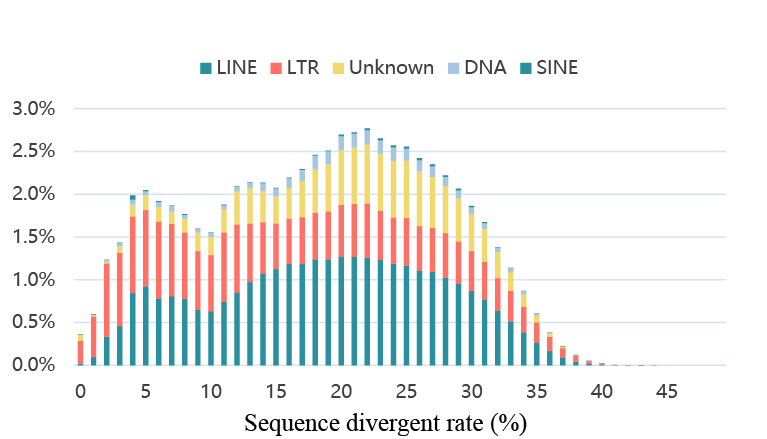

Supplement: S5 Fig — The classified transposon families in F. gigantica. (TIF) [file pntd.0009750.s005.tif]

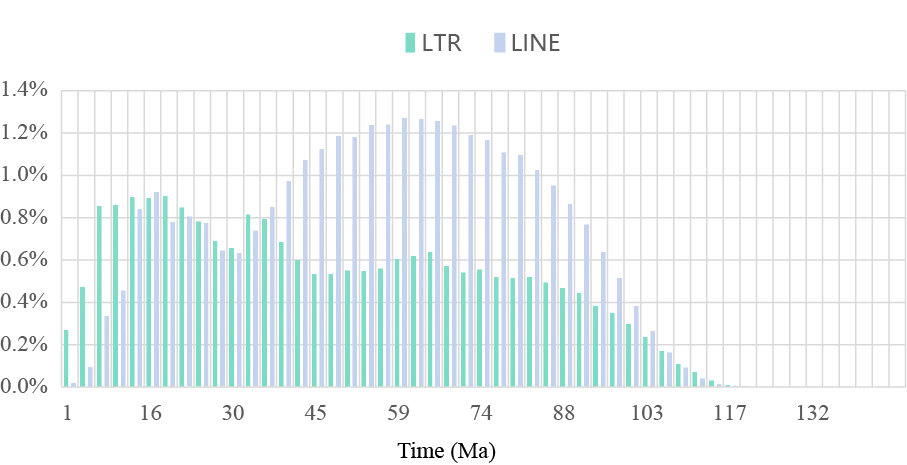

Supplement: S6 Fig — The mutation rate was 1.73×10^−9^. (TIF) [file pntd.0009750.s006.tif]

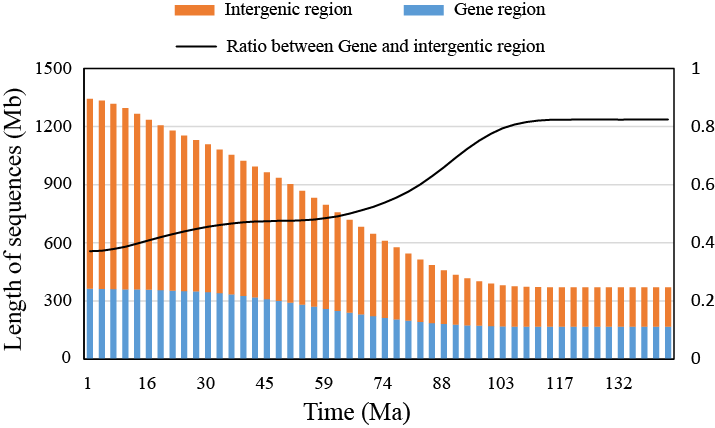

Supplement: S7 Fig — The mutation rate was 1.73×10^−9^. (TIF) [file pntd.0009750.s007.tif]

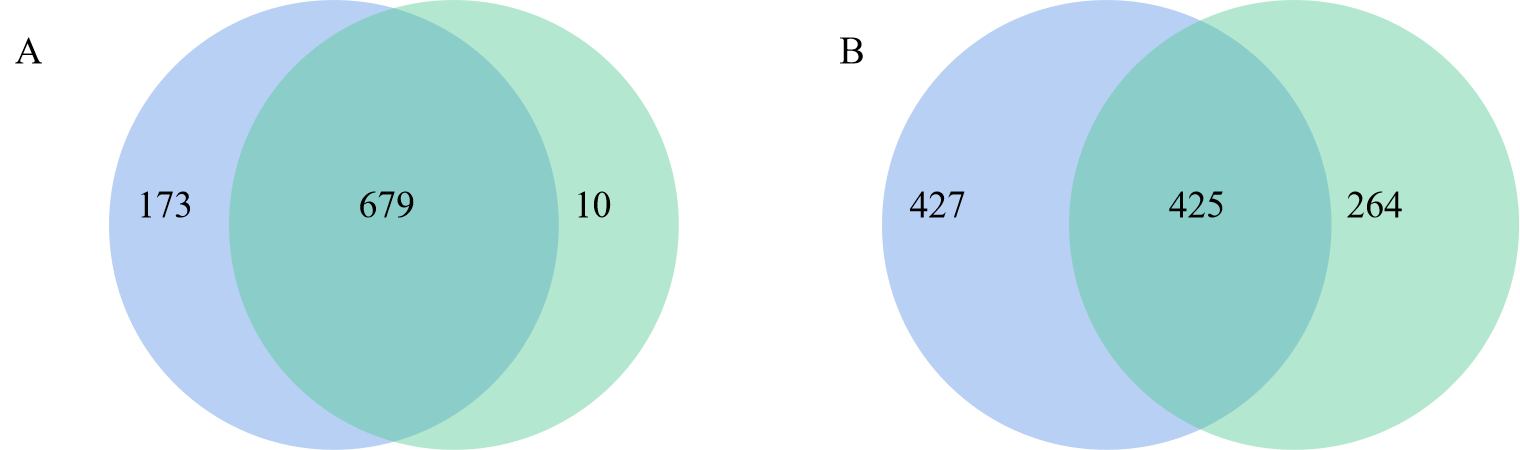

Supplement: S8 Fig — (TIF) [file pntd.0009750.s008.tif]
