# Supplementary material for: High-quality reference genome of Fasciola gigantica: Insights into the genomic signatures of transposon-mediated evolution and specific parasitic adaption in tropical regions
Source: PLoS Negl Trop Dis. 2021 Oct 5;15(10):e0009750. doi: 10.1371/journal.pntd.0009750 (PMC8519440; doi:10.1371/journal.pntd.0009750)
Supplement: S1 Text — (DOC) [file pntd.0009750.s026.doc]

group1 1 225320 1 W ctg00319 1 225320 -

group1 225321 225420 2 U 100 contig yes map

group1 225421 2057128 3 W ctg00058 1 1831708 +

group1 2057129 2057228 4 U 100 contig yes map

group1 2057229 7399232 5 W ctg00506 1 5342004 +

group1 7399233 7399332 6 U 100 contig yes map

group1 7399333 7860680 7 W ctg00723 1 461348 +

group1 7860681 7860780 8 U 100 contig yes map

group1 7860781 7947203 9 W ctg00606 1 86423 +

group1 7947204 7947303 10 U 100 contig yes map

group1 7947304 8335202 11 W ctg00485 1 387899 -

group1 8335203 8335302 12 U 100 contig yes map

group1 8335303 13045668 13 W ctg00992 1 4710366 +

group1 13045669 13045768 14 U 100 contig yes map

group1 13045769 13868473 15 W ctg00920 1 822705 +

group1 13868474 13868573 16 U 100 contig yes map

group1 13868574 14383072 17 W ctg00677 1 514499 +

group1 14383073 14383172 18 U 100 contig yes map

group1 14383173 14841415 19 W ctg00186 1 458243 -

group1 14841416 14841515 20 U 100 contig yes map

group1 14841516 15268980 21 W ctg00777 1 427465 +

group1 15268981 15269080 22 U 100 contig yes map

group1 15269081 15359022 23 W ctg00837 1 89942 +

group1 15359023 15359122 24 U 100 contig yes map

group1 15359123 23521791 25 W ctg00225 1 8162669 -

group1 23521792 23521891 26 U 100 contig yes map

group1 23521892 23562778 27 W ctg00854 1 40887 +

group1 23562779 23562878 28 U 100 contig yes map

group1 23562879 24563366 29 W ctg00010 1 1000488 -

group1 24563367 24563466 30 U 100 contig yes map

group1 24563467 25904691 31 W ctg00935 1 1341225 -

group1 25904692 25904791 32 U 100 contig yes map

group1 25904792 30797289 33 W ctg00635 1 4892498 -

group1 30797290 30797389 34 U 100 contig yes map

group1 30797390 30826547 35 W ctg00970 1 29158 -

group1 30826548 30826647 36 U 100 contig yes map

group1 30826648 36669063 37 W ctg00833 1 5842416 +

group1 36669064 36669163 38 U 100 contig yes map

group1 36669164 37378802 39 W ctg00559 1 709639 -

group1 37378803 37378902 40 U 100 contig yes map

group1 37378903 43448730 41 W ctg00682 1 6069828 -

group1 43448731 43448830 42 U 100 contig yes map

group1 43448831 43528555 43 W ctg01015 1 79725 -

group1 43528556 43528655 44 U 100 contig yes map

group1 43528656 43598030 45 W ctg00966 1 69375 -

group1 43598031 43598130 46 U 100 contig yes map

group1 43598131 43681312 47 W ctg00390 1 83182 +

group1 43681313 43681412 48 U 100 contig yes map

group1 43681413 43774573 49 W ctg00205 1 93161 -

group1 43774574 43774673 50 U 100 contig yes map

group1 43774674 45321256 51 W ctg00509 1 1546583 +

group1 45321257 45321356 52 U 100 contig yes map

group1 45321357 52852958 53 W ctg00122 1 7531602 +

group1 52852959 52853058 54 U 100 contig yes map

group1 52853059 52854770 55 W ctg00879 1 1712 +

group1 52854771 52854870 56 U 100 contig yes map

group1 52854871 62967138 57 W ctg00870 1 10112268 -

group1 62967139 62967238 58 U 100 contig yes map

group1 62967239 63061899 59 W ctg00033 1 94661 -

group1 63061900 63061999 60 U 100 contig yes map

group1 63062000 63116703 61 W ctg00963 1 54704 -

group1 63116704 63116803 62 U 100 contig yes map

group1 63116804 63179638 63 W ctg00732 1 62835 -

group1 63179639 63179738 64 U 100 contig yes map

group1 63179739 78055984 65 W ctg00166 1 14876246 -

group1 78055985 78056084 66 U 100 contig yes map

group1 78056085 78086919 67 W ctg00526 1 30835 +

group1 78086920 78087019 68 U 100 contig yes map

group1 78087020 82452593 69 W ctg00188 1 4365574 -

group1 82452594 82452693 70 U 100 contig yes map

group1 82452694 83036841 71 W ctg00487 1 584148 +

group1 83036842 83036941 72 U 100 contig yes map

group1 83036942 83130171 73 W ctg00609 1 93230 -

group1 83130172 83130271 74 U 100 contig yes map

group1 83130272 83730885 75 W ctg00965 1 600614 -

group1 83730886 83730985 76 U 100 contig yes map

group1 83730986 85838624 77 W ctg00611 1 2107639 -

group1 85838625 85838724 78 U 100 contig yes map

group1 85838725 92857261 79 W ctg00162 1 7018537 +

group1 92857262 92857361 80 U 100 contig yes map

group1 92857362 92943343 81 W ctg00080 1 85982 +

group1 92943344 92943443 82 U 100 contig yes map

group1 92943444 93088716 83 W ctg00776 1 145273 +

group1 93088717 93088816 84 U 100 contig yes map

group1 93088817 94015674 85 W ctg00362 1 926858 +

group1 94015675 94015774 86 U 100 contig yes map

group1 94015775 94243282 87 W ctg00908 1 227508 -

group1 94243283 94243382 88 U 100 contig yes map

group1 94243383 94324653 89 W ctg00352 1 81271 -

group1 94324654 94324753 90 U 100 contig yes map

group1 94324754 94425658 91 W ctg00803 1 100905 +

group1 94425659 94425758 92 U 100 contig yes map

group1 94425759 94905285 93 W ctg00316 1 479527 -

group1 94905286 94905385 94 U 100 contig yes map

group1 94905386 95311044 95 W ctg00977 1 405659 -

group1 95311045 95311144 96 U 100 contig yes map

group1 95311145 102249948 97 W ctg00618 1 6938804 +

group1 102249949 102250048 98 U 100 contig yes map

group1 102250049 113439043 99 W ctg00607 1 11188995 -

group1 113439044 113439143 100 U 100 contig yes map

group1 113439144 113453008 101 W ctg01030 1 13865 +

group1 113453009 113453108 102 U 100 contig yes map

group1 113453109 113582552 103 W ctg01027 1 129444 +

group1 113582553 113582652 104 U 100 contig yes map

group1 113582653 115782541 105 W ctg00323 1 2199889 +

group1 115782542 115782641 106 U 100 contig yes map

group1 115782642 120201618 107 W ctg00851 1 4418977 +

group1 120201619 120201718 108 U 100 contig yes map

group1 120201719 123691576 109 W ctg00856 1 3489858 +

group1 123691577 123691676 110 U 100 contig yes map

group1 123691677 124204676 111 W ctg00696 1 513000 +

group1 124204677 124204776 112 U 100 contig yes map

group1 124204777 126645918 113 W ctg00619 1 2441142 -

group1 126645919 126646018 114 U 100 contig yes map

group1 126646019 135644051 115 W ctg00789 1 8998033 -

group1 135644052 135644151 116 U 100 contig yes map

group1 135644152 137040002 117 W ctg00350 1 1395851 +

group1 137040003 137040102 118 U 100 contig yes map

group1 137040103 138817986 119 W ctg00443 1 1777884 +

group1 138817987 138818086 120 U 100 contig yes map

group1 138818087 145202105 121 W ctg00597 1 6384019 -

group1 145202106 145202205 122 U 100 contig yes map

group1 145202206 148706802 123 W ctg00894 1 3504597 -

group1 148706803 148706902 124 U 100 contig yes map

group1 148706903 152412864 125 W ctg00663 1 3705962 -

group1 152412865 152412964 126 U 100 contig yes map

group1 152412965 157512832 127 W ctg00552 1 5099868 +

group1 157512833 157512932 128 U 100 contig yes map

group1 157512933 157652549 129 W ctg00154 1 139617 -

group1 157652550 157652649 130 U 100 contig yes map

group1 157652650 157729446 131 W ctg00561 1 76797 -

group1 157729447 157729546 132 U 100 contig yes map

group1 157729547 160491908 133 W ctg00031 1 2762362 -

group1 160491909 160492008 134 U 100 contig yes map

group1 160492009 164274996 135 W ctg00867 1 3782988 +

group1 164274997 164275096 136 U 100 contig yes map

group1 164275097 165590978 137 W ctg00456 1 1315882 +

group1 165590979 165591078 138 U 100 contig yes map

group1 165591079 166357674 139 W ctg00153 1 766596 +

group1 166357675 166357774 140 U 100 contig yes map

group1 166357775 168132787 141 W ctg00249 1 1775013 +

group1 168132788 168132887 142 U 100 contig yes map

group1 168132888 180998881 143 W ctg00216 1 12865994 -

group1 180998882 180998981 144 U 100 contig yes map

group1 180998982 181055081 145 W ctg00312 1 56100 +

group1 181055082 181055181 146 U 100 contig yes map

group1 181055182 181056993 147 W ctg00053 1 1812 +

group1 181056994 181057093 148 U 100 contig yes map

group1 181057094 185005291 149 W ctg00518 1 3948198 -

group1 185005292 185005391 150 U 100 contig yes map

group1 185005392 185154004 151 W ctg00625 1 148613 +

group1 185154005 185154104 152 U 100 contig yes map

group1 185154105 185172304 153 W ctg00781 1 18200 -

group1 185172305 185172404 154 U 100 contig yes map

group1 185172405 192342776 155 W ctg00276 1 7170372 -

group1 192342777 192342876 156 U 100 contig yes map

group1 192342877 196307253 157 W ctg00309 1 3964377 -

group1 196307254 196307353 158 U 100 contig yes map

group1 196307354 196728280 159 W ctg00211 1 420927 -

group1 196728281 196728380 160 U 100 contig yes map

group1 196728381 197672665 161 W ctg00064 1 944285 -

group1 197672666 197672765 162 U 100 contig yes map

group1 197672766 200391741 163 W ctg00077 1 2718976 -

group1 200391742 200391841 164 U 100 contig yes map

group1 200391842 202750349 165 W ctg00768 1 2358508 -

group1 202750350 202750449 166 U 100 contig yes map

group1 202750450 203404656 167 W ctg00273 1 654207 +

group1 203404657 203404756 168 U 100 contig yes map

group1 203404757 203451965 169 W ctg00183 1 47209 -

group1 203451966 203452065 170 U 100 contig yes map

group1 203452066 208658659 171 W ctg00100 1 5206594 -

group1 208658660 208658759 172 U 100 contig yes map

group1 208658760 209669252 173 W ctg00403 1 1010493 -

group1 209669253 209669352 174 U 100 contig yes map

group1 209669353 210942189 175 W ctg00155 1 1272837 +

group1 210942190 210942289 176 U 100 contig yes map

group1 210942290 211038730 177 W ctg01029 1 96441 -

group1 211038731 211038830 178 U 100 contig yes map

group1 211038831 211149707 179 W ctg00052 1 110877 -

group1 211149708 211149807 180 U 100 contig yes map

group1 211149808 211352145 181 W ctg00861 1 202338 +

group1 211352146 211352245 182 U 100 contig yes map

group1 211352246 211409513 183 W ctg00698 1 57268 -

group1 211409514 211409613 184 U 100 contig yes map

group1 211409614 211981896 185 W ctg00313 1 572283 +

group1 211981897 211981996 186 U 100 contig yes map

group1 211981997 212647549 187 W ctg00133 1 665553 +

group1 212647550 212647649 188 U 100 contig yes map

group1 212647650 219145666 189 W ctg01000 1 6498017 +

group1 219145667 219145766 190 U 100 contig yes map

group1 219145767 221453126 191 W ctg00669 1 2307360 -

group1 221453127 221453226 192 U 100 contig yes map

group1 221453227 224509354 193 W ctg00246 1 3056128 +

group1 224509355 224509454 194 U 100 contig yes map

group1 224509455 233717103 195 W ctg00678 1 9207649 +

group1 233717104 233717203 196 U 100 contig yes map

group1 233717204 234916878 197 W ctg00567 1 1199675 +

group1 234916879 234916978 198 U 100 contig yes map

group1 234916979 241938672 199 W ctg00765 1 7021694 -

group1 241938673 241938772 200 U 100 contig yes map

group1 241938773 242930552 201 W ctg01025 1 991780 -

group1 242930553 242930652 202 U 100 contig yes map

group1 242930653 243118995 203 W ctg00167 1 188343 +

group1 243118996 243119095 204 U 100 contig yes map

group1 243119096 243357855 205 W ctg00144 1 238760 -

group1 243357856 243357955 206 U 100 contig yes map

group1 243357956 243360521 207 W ctg00565 1 2566 +

group1 243360522 243360621 208 U 100 contig yes map

group1 243360622 243500049 209 W ctg00384 1 139428 -

group1 243500050 243500149 210 U 100 contig yes map

group1 243500150 251705019 211 W ctg00157 1 8204870 -

group1 251705020 251705119 212 U 100 contig yes map

group1 251705120 251781840 213 W ctg00907 1 76721 +

group1 251781841 251781940 214 U 100 contig yes map

group1 251781941 252356389 215 W ctg00435 1 574449 -

group1 252356390 252356489 216 U 100 contig yes map

group1 252356490 253536496 217 W ctg00939 1 1180007 +

group1 253536497 253536596 218 U 100 contig yes map

group1 253536597 254001303 219 W ctg00948 1 464707 +

group1 254001304 254001403 220 U 100 contig yes map

group1 254001404 255933706 221 W ctg00389 1 1932303 +

group1 255933707 255933806 222 U 100 contig yes map

group1 255933807 259178423 223 W ctg00300 1 3244617 +

group1 259178424 259178523 224 U 100 contig yes map

group1 259178524 261111562 225 W ctg00513 1 1933039 -

group1 261111563 261111662 226 U 100 contig yes map

group1 261111663 261258408 227 W ctg00820 1 146746 -

group1 261258409 261258508 228 U 100 contig yes map

group1 261258509 262507195 229 W ctg00742 1 1248687 +

group1 262507196 262507295 230 U 100 contig yes map

group1 262507296 262544722 231 W ctg00274 1 37427 +

group1 262544723 262544822 232 U 100 contig yes map

group1 262544823 263392528 233 W ctg00982 1 847706 +

group1 263392529 263392628 234 U 100 contig yes map

group1 263392629 263845669 235 W ctg00099 1 453041 +

group1 263845670 263845769 236 U 100 contig yes map

group1 263845770 270730888 237 W ctg00890 1 6885119 +

group1 270730889 270730988 238 U 100 contig yes map

group1 270730989 270794655 239 W ctg00553 1 63667 +

group1 270794656 270794755 240 U 100 contig yes map

group1 270794756 270849163 241 W ctg00653 1 54408 -

group1 270849164 270849263 242 U 100 contig yes map

group1 270849264 270939689 243 W ctg00624 1 90426 -

group1 270939690 270939789 244 U 100 contig yes map

group1 270939790 289324387 245 W ctg00409 1 18384598 -

group10 1 238151 1 W ctg01021 1 238151 -

group10 238152 238251 2 U 100 contig yes map

group10 238252 11232198 3 W ctg00998 1 10993947 +

group10 11232199 11232298 4 U 100 contig yes map

group10 11232299 15466552 5 W ctg00439 1 4234254 +

group10 15466553 15466652 6 U 100 contig yes map

group10 15466653 18718936 7 W ctg01019 1 3252284 +

group10 18718937 18719036 8 U 100 contig yes map

group10 18719037 19912347 9 W ctg00078 1 1193311 +

group10 19912348 19912447 10 U 100 contig yes map

group10 19912448 21808749 11 W ctg00816 1 1896302 +

group10 21808750 21808849 12 U 100 contig yes map

group10 21808850 22340180 13 W ctg00873 1 531331 -

group10 22340181 22340280 14 U 100 contig yes map

group10 22340281 22828654 15 W ctg00087 1 488374 -

group10 22828655 22828754 16 U 100 contig yes map

group10 22828755 24042668 17 W ctg00724 1 1213914 -

group10 24042669 24042768 18 U 100 contig yes map

group10 24042769 24159724 19 W ctg00593 1 116956 +

group10 24159725 24159824 20 U 100 contig yes map

group10 24159825 26029748 21 W ctg00850 1 1869924 -

group10 26029749 26029848 22 U 100 contig yes map

group10 26029849 28589127 23 W ctg00960 1 2559279 -

group10 28589128 28589227 24 U 100 contig yes map

group10 28589228 29972319 25 W ctg00030 1 1383092 -

group10 29972320 29972419 26 U 100 contig yes map

group10 29972420 35098198 27 W ctg00896 1 5125779 +

group10 35098199 35098298 28 U 100 contig yes map

group10 35098299 35305363 29 W ctg00949 1 207065 -

group10 35305364 35305463 30 U 100 contig yes map

group10 35305464 35496039 31 W ctg00783 1 190576 +

group10 35496040 35496139 32 U 100 contig yes map

group10 35496140 35543923 33 W ctg00164 1 47784 -

group10 35543924 35544023 34 U 100 contig yes map

group10 35544024 35590456 35 W ctg00612 1 46433 -

group10 35590457 35590556 36 U 100 contig yes map

group10 35590557 35742419 37 W ctg00729 1 151863 -

group10 35742420 35742519 38 U 100 contig yes map

group10 35742520 35916002 39 W ctg00786 1 173483 -

group10 35916003 35916102 40 U 100 contig yes map

group10 35916103 36189420 41 W ctg00471 1 273318 -

group10 36189421 36189520 42 U 100 contig yes map

group10 36189521 36250372 43 W ctg00822 1 60852 -

group10 36250373 36250472 44 U 100 contig yes map

group10 36250473 39681779 45 W ctg00189 1 3431307 +

group10 39681780 39681879 46 U 100 contig yes map

group10 39681880 40265102 47 W ctg00095 1 583223 -

group10 40265103 40265202 48 U 100 contig yes map

group10 40265203 40294807 49 W ctg00283 1 29605 +

group10 40294808 40294907 50 U 100 contig yes map

group10 40294908 40614912 51 W ctg01001 1 320005 +

group10 40614913 40615012 52 U 100 contig yes map

group10 40615013 40694485 53 W ctg00503 1 79473 -

group10 40694486 40694585 54 U 100 contig yes map

group10 40694586 40838681 55 W ctg00730 1 144096 +

group10 40838682 40838781 56 U 100 contig yes map

group10 40838782 40971779 57 W ctg00734 1 132998 +

group10 40971780 40971879 58 U 100 contig yes map

group10 40971880 41139733 59 W ctg01018 1 167854 -

group10 41139734 41139833 60 U 100 contig yes map

group10 41139834 41233820 61 W ctg00028 1 93987 +

group10 41233821 41233920 62 U 100 contig yes map

group10 41233921 41884506 63 W ctg00772 1 650586 -

group10 41884507 41884606 64 U 100 contig yes map

group10 41884607 42225520 65 W ctg00661 1 340914 +

group10 42225521 42225620 66 U 100 contig yes map

group10 42225621 42427897 67 W ctg00744 1 202277 -

group10 42427898 42427997 68 U 100 contig yes map

group10 42427998 42477036 69 W ctg00512 1 49039 -

group10 42477037 42477136 70 U 100 contig yes map

group10 42477137 43003993 71 W ctg00930 1 526857 -

group10 43003994 43004093 72 U 100 contig yes map

group10 43004094 43432271 73 W ctg00168 1 428178 +

group10 43432272 43432371 74 U 100 contig yes map

group10 43432372 43724129 75 W ctg00647 1 291758 +

group10 43724130 43724229 76 U 100 contig yes map

group10 43724230 44576671 77 W ctg00322 1 852442 +

group10 44576672 44576771 78 U 100 contig yes map

group10 44576772 45042629 79 W ctg00057 1 465858 -

group10 45042630 45042729 80 U 100 contig yes map

group10 45042730 45336520 81 W ctg00408 1 293791 +

group10 45336521 45336620 82 U 100 contig yes map

group10 45336621 45719666 83 W ctg00008 1 383046 -

group10 45719667 45719766 84 U 100 contig yes map

group10 45719767 46075005 85 W ctg00990 1 355239 -

group10 46075006 46075105 86 U 100 contig yes map

group10 46075106 46096166 87 W ctg00697 1 21061 -

group10 46096167 46096266 88 U 100 contig yes map

group10 46096267 48345010 89 W ctg00548 1 2248744 +

group10 48345011 48345110 90 U 100 contig yes map

group10 48345111 48431724 91 W ctg00346 1 86614 -

group10 48431725 48431824 92 U 100 contig yes map

group10 48431825 48484678 93 W ctg00426 1 52854 -

group10 48484679 48484778 94 U 100 contig yes map

group10 48484779 48546189 95 W ctg00422 1 61411 -

group10 48546190 48546289 96 U 100 contig yes map

group10 48546290 48665585 97 W ctg00202 1 119296 +

group10 48665586 48665685 98 U 100 contig yes map

group10 48665686 48724245 99 W ctg00324 1 58560 +

group10 48724246 48724345 100 U 100 contig yes map

group10 48724346 48863079 101 W ctg00785 1 138734 +

group10 48863080 48863179 102 U 100 contig yes map

group10 48863180 49967824 103 W ctg00916 1 1104645 +

group10 49967825 49967924 104 U 100 contig yes map

group10 49967925 50049734 105 W ctg00794 1 81810 -

group10 50049735 50049834 106 U 100 contig yes map

group10 50049835 50184142 107 W ctg00891 1 134308 +

group10 50184143 50184242 108 U 100 contig yes map

group10 50184243 50273062 109 W ctg00131 1 88820 +

group10 50273063 50273162 110 U 100 contig yes map

group10 50273163 50352916 111 W ctg00290 1 79754 +

group10 50352917 50353016 112 U 100 contig yes map

group10 50353017 50427807 113 W ctg00123 1 74791 -

group10 50427808 50427907 114 U 100 contig yes map

group10 50427908 50506564 115 W ctg00001 1 78657 +

group10 50506565 50506664 116 U 100 contig yes map

group10 50506665 50586921 117 W ctg00497 1 80257 -

group10 50586922 50587021 118 U 100 contig yes map

group10 50587022 50638336 119 W ctg00627 1 51315 -

group10 50638337 50638436 120 U 100 contig yes map

group10 50638437 50853065 121 W ctg00222 1 214629 -

group10 50853066 50853165 122 U 100 contig yes map

group10 50853166 50940972 123 W ctg00092 1 87807 +

group10 50940973 50941072 124 U 100 contig yes map

group10 50941073 51042814 125 W ctg00494 1 101742 +

group2 1 58224 1 W ctg00296 1 58224 -

group2 58225 58324 2 U 100 contig yes map

group2 58325 114173 3 W ctg00267 1 55849 -

group2 114174 114273 4 U 100 contig yes map

group2 114274 200527 5 W ctg00847 1 86254 +

group2 200528 200627 6 U 100 contig yes map

group2 200628 269242 7 W ctg00438 1 68615 +

group2 269243 269342 8 U 100 contig yes map

group2 269343 365564 9 W ctg00451 1 96222 +

group2 365565 365664 10 U 100 contig yes map

group2 365665 448493 11 W ctg00802 1 82829 -

group2 448494 448593 12 U 100 contig yes map

group2 448594 5507809 13 W ctg00979 1 5059216 -

group2 5507810 5507909 14 U 100 contig yes map

group2 5507910 5614637 15 W ctg00616 1 106728 -

group2 5614638 5614737 16 U 100 contig yes map

group2 5614738 6564461 17 W ctg00554 1 949724 +

group2 6564462 6564561 18 U 100 contig yes map

group2 6564562 7684929 19 W ctg00795 1 1120368 -

group2 7684930 7685029 20 U 100 contig yes map

group2 7685030 7756053 21 W ctg00672 1 71024 -

group2 7756054 7756153 22 U 100 contig yes map

group2 7756154 7808337 23 W ctg00866 1 52184 -

group2 7808338 7808437 24 U 100 contig yes map

group2 7808438 7877962 25 W ctg00751 1 69525 -

group2 7877963 7878062 26 U 100 contig yes map

group2 7878063 7946902 27 W ctg01024 1 68840 -

group2 7946903 7947002 28 U 100 contig yes map

group2 7947003 8030955 29 W ctg00357 1 83953 -

group2 8030956 8031055 30 U 100 contig yes map

group2 8031056 8088837 31 W ctg00111 1 57782 -

group2 8088838 8088937 32 U 100 contig yes map

group2 8088938 8157279 33 W ctg00507 1 68342 -

group2 8157280 8157379 34 U 100 contig yes map

group2 8157380 8250497 35 W ctg00790 1 93118 -

group2 8250498 8250597 36 U 100 contig yes map

group2 8250598 8253715 37 W ctg00871 1 3118 +

group2 8253716 8253815 38 U 100 contig yes map

group2 8253816 8421742 39 W ctg00865 1 167927 +

group2 8421743 8421842 40 U 100 contig yes map

group2 8421843 9415224 41 W ctg00041 1 993382 -

group2 9415225 9415324 42 U 100 contig yes map

group2 9415325 9771632 43 W ctg00859 1 356308 -

group2 9771633 9771732 44 U 100 contig yes map

group2 9771733 9907230 45 W ctg00253 1 135498 +

group2 9907231 9907330 46 U 100 contig yes map

group2 9907331 9969429 47 W ctg00232 1 62099 -

group2 9969430 9969529 48 U 100 contig yes map

group2 9969530 11660619 49 W ctg00594 1 1691090 +

group2 11660620 11660719 50 U 100 contig yes map

group2 11660720 12441673 51 W ctg00479 1 780954 -

group2 12441674 12441773 52 U 100 contig yes map

group2 12441774 13377681 53 W ctg00335 1 935908 -

group2 13377682 13377781 54 U 100 contig yes map

group2 13377782 16638121 55 W ctg00504 1 3260340 +

group2 16638122 16638221 56 U 100 contig yes map

group2 16638222 24802696 57 W ctg00165 1 8164475 -

group2 24802697 24802796 58 U 100 contig yes map

group2 24802797 25064923 59 W ctg00800 1 262127 +

group2 25064924 25065023 60 U 100 contig yes map

group2 25065024 25244441 61 W ctg00387 1 179418 +

group2 25244442 25244541 62 U 100 contig yes map

group2 25244542 25670981 63 W ctg00710 1 426440 +

group2 25670982 25671081 64 U 100 contig yes map

group2 25671082 33697661 65 W ctg00976 1 8026580 +

group2 33697662 33697761 66 U 100 contig yes map

group2 33697762 35775073 67 W ctg00922 1 2077312 -

group2 35775074 35775173 68 U 100 contig yes map

group2 35775174 36268591 69 W ctg00717 1 493418 -

group2 36268592 36268691 70 U 100 contig yes map

group2 36268692 37727521 71 W ctg01020 1 1458830 +

group2 37727522 37727621 72 U 100 contig yes map

group2 37727622 44405731 73 W ctg00478 1 6678110 -

group2 44405732 44405831 74 U 100 contig yes map

group2 44405832 49840129 75 W ctg00993 1 5434298 +

group2 49840130 49840229 76 U 100 contig yes map

group2 49840230 54439391 77 W ctg00906 1 4599162 +

group2 54439392 54439491 78 U 100 contig yes map

group2 54439492 54813109 79 W ctg00235 1 373618 +

group2 54813110 54813209 80 U 100 contig yes map

group2 54813210 56063834 81 W ctg00226 1 1250625 +

group2 56063835 56063934 82 U 100 contig yes map

group2 56063935 59805140 83 W ctg00892 1 3741206 +

group2 59805141 59805240 84 U 100 contig yes map

group2 59805241 67767918 85 W ctg00748 1 7962678 +

group2 67767919 67768018 86 U 100 contig yes map

group2 67768019 70851754 87 W ctg00615 1 3083736 -

group2 70851755 70851854 88 U 100 contig yes map

group2 70851855 77398701 89 W ctg00702 1 6546847 -

group2 77398702 77398801 90 U 100 contig yes map

group2 77398802 77459935 91 W ctg00605 1 61134 -

group2 77459936 77460035 92 U 100 contig yes map

group2 77460036 78971920 93 W ctg00063 1 1511885 -

group2 78971921 78972020 94 U 100 contig yes map

group2 78972021 79077790 95 W ctg00302 1 105770 -

group2 79077791 79077890 96 U 100 contig yes map

group2 79077891 79128928 97 W ctg00492 1 51038 -

group2 79128929 79129028 98 U 100 contig yes map

group2 79129029 80369693 99 W ctg00179 1 1240665 +

group2 80369694 80369793 100 U 100 contig yes map

group2 80369794 80669607 101 W ctg00489 1 299814 +

group2 80669608 80669707 102 U 100 contig yes map

group2 80669708 80740399 103 W ctg00004 1 70692 +

group2 80740400 80740499 104 U 100 contig yes map

group2 80740500 80800090 105 W ctg00148 1 59591 +

group2 80800091 80800190 106 U 100 contig yes map

group2 80800191 90711375 107 W ctg00522 1 9911185 -

group2 90711376 90711475 108 U 100 contig yes map

group2 90711476 91761792 109 W ctg00546 1 1050317 +

group2 91761793 91761892 110 U 100 contig yes map

group2 91761893 93511700 111 W ctg00934 1 1749808 +

group2 93511701 93511800 112 U 100 contig yes map

group2 93511801 95037804 113 W ctg00757 1 1526004 +

group2 95037805 95037904 114 U 100 contig yes map

group2 95037905 95186255 115 W ctg00600 1 148351 +

group2 95186256 95186355 116 U 100 contig yes map

group2 95186356 100986708 117 W ctg00670 1 5800353 -

group2 100986709 100986808 118 U 100 contig yes map

group2 100986809 108005999 119 W ctg00161 1 7019191 -

group2 108006000 108006099 120 U 100 contig yes map

group2 108006100 108056539 121 W ctg00756 1 50440 -

group2 108056540 108056639 122 U 100 contig yes map

group2 108056640 117435757 123 W ctg00840 1 9379118 +

group2 117435758 117435857 124 U 100 contig yes map

group2 117435858 117560932 125 W ctg00040 1 125075 -

group2 117560933 117561032 126 U 100 contig yes map

group2 117561033 117652045 127 W ctg00248 1 91013 +

group2 117652046 117652145 128 U 100 contig yes map

group2 117652146 117733606 129 W ctg00749 1 81461 -

group2 117733607 117733706 130 U 100 contig yes map

group2 117733707 119368710 131 W ctg00347 1 1635004 -

group2 119368711 119368810 132 U 100 contig yes map

group2 119368811 122751864 133 W ctg00843 1 3383054 +

group2 122751865 122751964 134 U 100 contig yes map

group2 122751965 123123394 135 W ctg00731 1 371430 +

group2 123123395 123123494 136 U 100 contig yes map

group2 123123495 123210067 137 W ctg00726 1 86573 -

group2 123210068 123210167 138 U 100 contig yes map

group2 123210168 123213409 139 W ctg00750 1 3242 -

group2 123213410 123213509 140 U 100 contig yes map

group2 123213510 123685986 141 W ctg00701 1 472477 +

group2 123685987 123686086 142 U 100 contig yes map

group2 123686087 133194875 143 W ctg00256 1 9508789 -

group2 133194876 133194975 144 U 100 contig yes map

group2 133194976 133285115 145 W ctg00521 1 90140 -

group2 133285116 133285215 146 U 100 contig yes map

group2 133285216 133336768 147 W ctg00580 1 51553 +

group2 133336769 133336868 148 U 100 contig yes map

group2 133336869 133378874 149 W ctg00093 1 42006 -

group2 133378875 133378974 150 U 100 contig yes map

group2 133378975 133507391 151 W ctg00024 1 128417 +

group2 133507392 133507491 152 U 100 contig yes map

group2 133507492 133656531 153 W ctg00455 1 149040 +

group2 133656532 133656631 154 U 100 contig yes map

group2 133656632 133689279 155 W ctg00367 1 32648 -

group2 133689280 133689379 156 U 100 contig yes map

group2 133689380 133734264 157 W ctg00973 1 44885 -

group2 133734265 133734364 158 U 100 contig yes map

group2 133734365 133778277 159 W ctg00307 1 43913 -

group2 133778278 133778377 160 U 100 contig yes map

group2 133778378 133844246 161 W ctg00972 1 65869 -

group2 133844247 133844346 162 U 100 contig yes map

group2 133844347 133897271 163 W ctg00417 1 52925 -

group2 133897272 133897371 164 U 100 contig yes map

group2 133897372 133954735 165 W ctg00573 1 57364 -

group2 133954736 133954835 166 U 100 contig yes map

group2 133954836 133997534 167 W ctg00017 1 42699 +

group2 133997535 133997634 168 U 100 contig yes map

group2 133997635 134045660 169 W ctg00835 1 48026 -

group2 134045661 134045760 170 U 100 contig yes map

group2 134045761 134106880 171 W ctg00563 1 61120 +

group2 134106881 134106980 172 U 100 contig yes map

group2 134106981 134185931 173 W ctg00196 1 78951 -

group2 134185932 134186031 174 U 100 contig yes map

group2 134186032 134277757 175 W ctg00575 1 91726 +

group2 134277758 134277857 176 U 100 contig yes map

group2 134277858 134334275 177 W ctg00441 1 56418 -

group2 134334276 134334375 178 U 100 contig yes map

group2 134334376 134374552 179 W ctg00280 1 40177 +

group2 134374553 134374652 180 U 100 contig yes map

group2 134374653 134410083 181 W ctg00761 1 35431 -

group2 134410084 134410183 182 U 100 contig yes map

group2 134410184 134456575 183 W ctg00914 1 46392 +

group2 134456576 134456675 184 U 100 contig yes map

group2 134456676 134531993 185 W ctg00969 1 75318 -

group2 134531994 134532093 186 U 100 contig yes map

group2 134532094 139185236 187 W ctg00536 1 4653143 -

group2 139185237 139185336 188 U 100 contig yes map

group2 139185337 139243680 189 W ctg00639 1 58344 -

group2 139243681 139243780 190 U 100 contig yes map

group2 139243781 139288575 191 W ctg00043 1 44795 -

group2 139288576 139288675 192 U 100 contig yes map

group2 139288676 139372064 193 W ctg00096 1 83389 +

group2 139372065 139372164 194 U 100 contig yes map

group2 139372165 139499220 195 W ctg00105 1 127056 -

group2 139499221 139499320 196 U 100 contig yes map

group2 139499321 141128407 197 W ctg00807 1 1629087 -

group2 141128408 141128507 198 U 100 contig yes map

group2 141128508 145116537 199 W ctg00013 1 3988030 -

group2 145116538 145116637 200 U 100 contig yes map

group2 145116638 145145116 201 W ctg00386 1 28479 +

group2 145145117 145145216 202 U 100 contig yes map

group2 145145217 145172729 203 W ctg00853 1 27513 +

group2 145172730 145172829 204 U 100 contig yes map

group2 145172830 145227504 205 W ctg00858 1 54675 +

group2 145227505 145227604 206 U 100 contig yes map

group2 145227605 145549330 207 W ctg00353 1 321726 +

group2 145549331 145549430 208 U 100 contig yes map

group2 145549431 145611724 209 W ctg00699 1 62294 +

group2 145611725 145611824 210 U 100 contig yes map

group2 145611825 147860338 211 W ctg00869 1 2248514 +

group2 147860339 147860438 212 U 100 contig yes map

group2 147860439 150467911 213 W ctg00925 1 2607473 -

group2 150467912 150468011 214 U 100 contig yes map

group2 150468012 151813588 215 W ctg00282 1 1345577 -

group2 151813589 151813688 216 U 100 contig yes map

group2 151813689 153388711 217 W ctg00524 1 1575023 -

group2 153388712 153388811 218 U 100 contig yes map

group2 153388812 156814585 219 W ctg00944 1 3425774 -

group2 156814586 156814685 220 U 100 contig yes map

group2 156814686 156915914 221 W ctg00060 1 101229 +

group2 156915915 156916014 222 U 100 contig yes map

group2 156916015 157003093 223 W ctg00620 1 87079 +

group2 157003094 157003193 224 U 100 contig yes map

group2 157003194 157043865 225 W ctg00254 1 40672 -

group2 157043866 157043965 226 U 100 contig yes map

group2 157043966 157220949 227 W ctg00571 1 176984 -

group2 157220950 157221049 228 U 100 contig yes map

group2 157221050 157294893 229 W ctg00469 1 73844 -

group2 157294894 157294993 230 U 100 contig yes map

group2 157294994 158572823 231 W ctg01003 1 1277830 -

group2 158572824 158572923 232 U 100 contig yes map

group2 158572924 160336533 233 W ctg00012 1 1763610 +

group2 160336534 160336633 234 U 100 contig yes map

group2 160336634 160393049 235 W ctg00475 1 56416 -

group2 160393050 160393149 236 U 100 contig yes map

group2 160393150 161087872 237 W ctg01028 1 694723 +

group2 161087873 161087972 238 U 100 contig yes map

group2 161087973 161142829 239 W ctg00706 1 54857 +

group2 161142830 161142929 240 U 100 contig yes map

group2 161142930 161310762 241 W ctg00467 1 167833 -

group2 161310763 161310862 242 U 100 contig yes map

group2 161310863 161329288 243 W ctg00810 1 18426 -

group2 161329289 161329388 244 U 100 contig yes map

group2 161329389 161495055 245 W ctg00176 1 165667 -

group2 161495056 161495155 246 U 100 contig yes map

group2 161495156 161554853 247 W ctg00895 1 59698 +

group2 161554854 161554953 248 U 100 contig yes map

group2 161554954 163296964 249 W ctg00279 1 1742011 -

group2 163296965 163297064 250 U 100 contig yes map

group2 163297065 165531616 251 W ctg00239 1 2234552 +

group2 165531617 165531716 252 U 100 contig yes map

group2 165531717 172740346 253 W ctg00981 1 7208630 +

group2 172740347 172740446 254 U 100 contig yes map

group2 172740447 176526850 255 W ctg00498 1 3786404 -

group2 176526851 176526950 256 U 100 contig yes map

group2 176526951 176571679 257 W ctg00261 1 44729 -

group2 176571680 176571779 258 U 100 contig yes map

group2 176571780 176605489 259 W ctg00474 1 33710 -

group2 176605490 176605589 260 U 100 contig yes map

group2 176605590 176687562 261 W ctg00190 1 81973 -

group3 1 20797440 1 W ctg00203 1 20797440 -

group3 20797441 20797540 2 U 100 contig yes map

group3 20797541 20876539 3 W ctg00268 1 78999 -

group3 20876540 20876639 4 U 100 contig yes map

group3 20876640 20958325 5 W ctg00826 1 81686 +

group3 20958326 20958425 6 U 100 contig yes map

group3 20958426 21030769 7 W ctg00440 1 72344 -

group3 21030770 21030869 8 U 100 contig yes map

group3 21030870 21092219 9 W ctg00401 1 61350 -

group3 21092220 21092319 10 U 100 contig yes map

group3 21092320 21159256 11 W ctg00269 1 66937 +

group3 21159257 21159356 12 U 100 contig yes map

group3 21159357 27748587 13 W ctg00037 1 6589231 -

group3 27748588 27748687 14 U 100 contig yes map

group3 27748688 27782854 15 W ctg00442 1 34167 +

group3 27782855 27782954 16 U 100 contig yes map

group3 27782955 27814328 17 W ctg00529 1 31374 +

group3 27814329 27814428 18 U 100 contig yes map

group3 27814429 28412234 19 W ctg00753 1 597806 -

group3 28412235 28412334 20 U 100 contig yes map

group3 28412335 28489601 21 W ctg00360 1 77267 -

group3 28489602 28489701 22 U 100 contig yes map

group3 28489702 31038554 23 W ctg00463 1 2548853 +

group3 31038555 31038654 24 U 100 contig yes map

group3 31038655 35473350 25 W ctg00956 1 4434696 -

group3 35473351 35473450 26 U 100 contig yes map

group3 35473451 35704024 27 W ctg00634 1 230574 -

group3 35704025 35704124 28 U 100 contig yes map

group3 35704125 36450211 29 W ctg00827 1 746087 +

group3 36450212 36450311 30 U 100 contig yes map

group3 36450312 45824352 31 W ctg00755 1 9374041 -

group3 45824353 45824452 32 U 100 contig yes map

group3 45824453 55275784 33 W ctg00996 1 9451332 -

group3 55275785 55275884 34 U 100 contig yes map

group3 55275885 59272917 35 W ctg00120 1 3997033 -

group3 59272918 59273017 36 U 100 contig yes map

group3 59273018 61583642 37 W ctg00587 1 2310625 +

group3 61583643 61583742 38 U 100 contig yes map

group3 61583743 61657460 39 W ctg00023 1 73718 +

group3 61657461 61657560 40 U 100 contig yes map

group3 61657561 61746899 41 W ctg00792 1 89339 -

group3 61746900 61746999 42 U 100 contig yes map

group3 61747000 62842371 43 W ctg00009 1 1095372 -

group3 62842372 62842471 44 U 100 contig yes map

group3 62842472 68298116 45 W ctg00490 1 5455645 +

group3 68298117 68298216 46 U 100 contig yes map

group3 68298217 68358731 47 W ctg00713 1 60515 +

group3 68358732 68358831 48 U 100 contig yes map

group3 68358832 69507002 49 W ctg00903 1 1148171 -

group3 69507003 69507102 50 U 100 contig yes map

group3 69507103 69578586 51 W ctg00603 1 71484 -

group3 69578587 69578686 52 U 100 contig yes map

group3 69578687 72149302 53 W ctg00458 1 2570616 +

group3 72149303 72149402 54 U 100 contig yes map

group3 72149403 72410370 55 W ctg00595 1 260968 +

group3 72410371 72410470 56 U 100 contig yes map

group3 72410471 72493822 57 W ctg00917 1 83352 -

group3 72493823 72493922 58 U 100 contig yes map

group3 72493923 73029701 59 W ctg00195 1 535779 +

group3 73029702 73029801 60 U 100 contig yes map

group3 73029802 73091038 61 W ctg00825 1 61237 +

group3 73091039 73091138 62 U 100 contig yes map

group3 73091139 73638639 63 W ctg00086 1 547501 -

group3 73638640 73638739 64 U 100 contig yes map

group3 73638740 73827984 65 W ctg00788 1 189245 -

group3 73827985 73828084 66 U 100 contig yes map

group3 73828085 74205977 67 W ctg00975 1 377893 +

group3 74205978 74206077 68 U 100 contig yes map

group3 74206078 74907026 69 W ctg00549 1 700949 +

group3 74907027 74907126 70 U 100 contig yes map

group3 74907127 74998428 71 W ctg00234 1 91302 -

group3 74998429 74998528 72 U 100 contig yes map

group3 74998529 76667835 73 W ctg00187 1 1669307 +

group3 76667836 76667935 74 U 100 contig yes map

group3 76667936 76743988 75 W ctg00739 1 76053 +

group3 76743989 76744088 76 U 100 contig yes map

group3 76744089 76819500 77 W ctg00940 1 75412 +

group3 76819501 76819600 78 U 100 contig yes map

group3 76819601 78697016 79 W ctg00174 1 1877416 +

group3 78697017 78697116 80 U 100 contig yes map

group3 78697117 81469628 81 W ctg00844 1 2772512 +

group3 81469629 81469728 82 U 100 contig yes map

group3 81469729 82215142 83 W ctg00228 1 745414 +

group3 82215143 82215242 84 U 100 contig yes map

group3 82215243 82665073 85 W ctg00602 1 449831 +

group3 82665074 82665173 86 U 100 contig yes map

group3 82665174 84880910 87 W ctg00299 1 2215737 +

group3 84880911 84881010 88 U 100 contig yes map

group3 84881011 85023727 89 W ctg00900 1 142717 +

group3 85023728 85023827 90 U 100 contig yes map

group3 85023828 86610716 91 W ctg00666 1 1586889 -

group3 86610717 86610816 92 U 100 contig yes map

group3 86610817 88479145 93 W ctg00114 1 1868329 +

group3 88479146 88479245 94 U 100 contig yes map

group3 88479246 88539025 95 W ctg00264 1 59780 -

group3 88539026 88539125 96 U 100 contig yes map

group3 88539126 88830909 97 W ctg00623 1 291784 +

group3 88830910 88831009 98 U 100 contig yes map

group3 88831010 88868515 99 W ctg00361 1 37506 +

group3 88868516 88868615 100 U 100 contig yes map

group3 88868616 89086385 101 W ctg00709 1 217770 -

group3 89086386 89086485 102 U 100 contig yes map

group3 89086486 89121873 103 W ctg00688 1 35388 -

group3 89121874 89121973 104 U 100 contig yes map

group3 89121974 90840970 105 W ctg00088 1 1718997 +

group3 90840971 90841070 106 U 100 contig yes map

group3 90841071 91953295 107 W ctg00425 1 1112225 +

group3 91953296 91953395 108 U 100 contig yes map

group3 91953396 92033712 109 W ctg00255 1 80317 -

group3 92033713 92033812 110 U 100 contig yes map

group3 92033813 92119981 111 W ctg00405 1 86169 -

group3 92119982 92120081 112 U 100 contig yes map

group3 92120082 92174663 113 W ctg00617 1 54582 -

group3 92174664 92174763 114 U 100 contig yes map

group3 92174764 92256875 115 W ctg00855 1 82112 +

group3 92256876 92256975 116 U 100 contig yes map

group3 92256976 92315754 117 W ctg00520 1 58779 -

group3 92315755 92315854 118 U 100 contig yes map

group3 92315855 92421091 119 W ctg00676 1 105237 +

group3 92421092 92421191 120 U 100 contig yes map

group3 92421192 92483382 121 W ctg00576 1 62191 -

group3 92483383 92483482 122 U 100 contig yes map

group3 92483483 92933733 123 W ctg00178 1 450251 -

group3 92933734 92933833 124 U 100 contig yes map

group3 92933834 93922258 125 W ctg00432 1 988425 +

group3 93922259 93922358 126 U 100 contig yes map

group3 93922359 98642735 127 W ctg00159 1 4720377 -

group3 98642736 98642835 128 U 100 contig yes map

group3 98642836 108403229 129 W ctg00163 1 9760394 -

group3 108403230 108403329 130 U 100 contig yes map

group3 108403330 117897431 131 W ctg00355 1 9494102 -

group3 117897432 117897531 132 U 100 contig yes map

group3 117897532 118649561 133 W ctg00056 1 752030 +

group3 118649562 118649661 134 U 100 contig yes map

group3 118649662 119089075 135 W ctg00339 1 439414 +

group3 119089076 119089175 136 U 100 contig yes map

group3 119089176 119195540 137 W ctg00220 1 106365 -

group3 119195541 119195640 138 U 100 contig yes map

group3 119195641 122144339 139 W ctg00250 1 2948699 +

group3 122144340 122144439 140 U 100 contig yes map

group3 122144440 122204475 141 W ctg00303 1 60036 -

group3 122204476 122204575 142 U 100 contig yes map

group3 122204576 126448529 143 W ctg00022 1 4243954 +

group3 126448530 126448629 144 U 100 contig yes map

group3 126448630 127203840 145 W ctg00787 1 755211 +

group3 127203841 127203940 146 U 100 contig yes map

group3 127203941 127571164 147 W ctg00265 1 367224 -

group3 127571165 127571264 148 U 100 contig yes map

group3 127571265 127797604 149 W ctg00331 1 226340 -

group3 127797605 127797704 150 U 100 contig yes map

group3 127797705 127872928 151 W ctg00213 1 75224 -

group3 127872929 127873028 152 U 100 contig yes map

group3 127873029 137673391 153 W ctg00931 1 9800363 -

group3 137673392 137673491 154 U 100 contig yes map

group3 137673492 137732809 155 W ctg00446 1 59318 -

group3 137732810 137732909 156 U 100 contig yes map

group3 137732910 137930991 157 W ctg00572 1 198082 +

group3 137930992 137931091 158 U 100 contig yes map

group3 137931092 138380231 159 W ctg00823 1 449140 +

group3 138380232 138380331 160 U 100 contig yes map

group3 138380332 142142385 161 W ctg00156 1 3762054 +

group3 142142386 142142485 162 U 100 contig yes map

group3 142142486 142220910 163 W ctg00645 1 78425 +

group3 142220911 142221010 164 U 100 contig yes map

group3 142221011 142844588 165 W ctg00638 1 623578 +

group3 142844589 142844688 166 U 100 contig yes map

group3 142844689 144690900 167 W ctg00457 1 1846212 -

group3 144690901 144691000 168 U 100 contig yes map

group3 144691001 145866065 169 W ctg00107 1 1175065 +

group3 145866066 145866165 170 U 100 contig yes map

group3 145866166 152209456 171 W ctg00180 1 6343291 +

group3 152209457 152209556 172 U 100 contig yes map

group3 152209557 158194002 173 W ctg00292 1 5984446 +

group3 158194003 158194102 174 U 100 contig yes map

group3 158194103 158680170 175 W ctg00208 1 486068 +

group3 158680171 158680270 176 U 100 contig yes map

group3 158680271 158750128 177 W ctg00704 1 69858 +

group3 158750129 158750228 178 U 100 contig yes map

group3 158750229 158845794 179 W ctg00628 1 95566 +

group3 158845795 158845894 180 U 100 contig yes map

group3 158845895 158946303 181 W ctg00071 1 100409 +

group3 158946304 158946403 182 U 100 contig yes map

group3 158946404 159036041 183 W ctg00059 1 89638 -

group4 1 77582 1 W ctg00128 1 77582 -

group4 77583 77682 2 U 100 contig yes map

group4 77683 6400479 3 W ctg00769 1 6322797 +

group4 6400480 6400579 4 U 100 contig yes map

group4 6400580 10263641 5 W ctg00005 1 3863062 +

group4 10263642 10263741 6 U 100 contig yes map

group4 10263742 10300198 7 W ctg00377 1 36457 +

group4 10300199 10300298 8 U 100 contig yes map

group4 10300299 10708327 9 W ctg00622 1 408029 -

group4 10708328 10708427 10 U 100 contig yes map

group4 10708428 15440958 11 W ctg00818 1 4732531 +

group4 15440959 15441058 12 U 100 contig yes map

group4 15441059 16047884 13 W ctg00081 1 606826 +

group4 16047885 16047984 14 U 100 contig yes map

group4 16047985 16317440 15 W ctg00902 1 269456 +

group4 16317441 16317540 16 U 100 contig yes map

group4 16317541 17340873 17 W ctg00599 1 1023333 +

group4 17340874 17340973 18 U 100 contig yes map

group4 17340974 19506543 19 W ctg00889 1 2165570 -

group4 19506544 19506643 20 U 100 contig yes map

group4 19506644 20086548 21 W ctg00182 1 579905 -

group4 20086549 20086648 22 U 100 contig yes map

group4 20086649 20352288 23 W ctg00988 1 265640 +

group4 20352289 20352388 24 U 100 contig yes map

group4 20352389 20369585 25 W ctg00002 1 17197 +

group4 20369586 20369685 26 U 100 contig yes map

group4 20369686 20466188 27 W ctg00215 1 96503 +

group4 20466189 20466288 28 U 100 contig yes map

group4 20466289 20815287 29 W ctg00660 1 348999 +

group4 20815288 20815387 30 U 100 contig yes map

group4 20815388 20990543 31 W ctg00185 1 175156 +

group4 20990544 20990643 32 U 100 contig yes map

group4 20990644 21159928 33 W ctg00062 1 169285 +

group4 21159929 21160028 34 U 100 contig yes map

group4 21160029 21231143 35 W ctg00392 1 71115 +

group4 21231144 21231243 36 U 100 contig yes map

group4 21231244 22429315 37 W ctg00775 1 1198072 +

group4 22429316 22429415 38 U 100 contig yes map

group4 22429416 22553808 39 W ctg01010 1 124393 +

group4 22553809 22553908 40 U 100 contig yes map

group4 22553909 23428703 41 W ctg00872 1 874795 +

group4 23428704 23428803 42 U 100 contig yes map

group4 23428804 25739148 43 W ctg00584 1 2310345 +

group4 25739149 25739248 44 U 100 contig yes map

group4 25739249 26855899 45 W ctg00681 1 1116651 +

group4 26855900 26855999 46 U 100 contig yes map

group4 26856000 27458800 47 W ctg00577 1 602801 +

group4 27458801 27458900 48 U 100 contig yes map

group4 27458901 28283215 49 W ctg00285 1 824315 -

group4 28283216 28283315 50 U 100 contig yes map

group4 28283316 28373548 51 W ctg00680 1 90233 -

group4 28373549 28373648 52 U 100 contig yes map

group4 28373649 33919766 53 W ctg00098 1 5546118 -

group4 33919767 33919866 54 U 100 contig yes map

group4 33919867 35048725 55 W ctg00780 1 1128859 -

group4 35048726 35048825 56 U 100 contig yes map

group4 35048826 35554542 57 W ctg00370 1 505717 +

group4 35554543 35554642 58 U 100 contig yes map

group4 35554643 39374602 59 W ctg00691 1 3819960 -

group4 39374603 39374702 60 U 100 contig yes map

group4 39374703 39404779 61 W ctg00224 1 30077 +

group4 39404780 39404879 62 U 100 contig yes map

group4 39404880 39504254 63 W ctg00271 1 99375 +

group4 39504255 39504354 64 U 100 contig yes map

group4 39504355 39530501 65 W ctg00656 1 26147 -

group4 39530502 39530601 66 U 100 contig yes map

group4 39530602 39589990 67 W ctg00655 1 59389 -

group4 39589991 39590090 68 U 100 contig yes map

group4 39590091 39650515 69 W ctg00649 1 60425 -

group4 39650516 39650615 70 U 100 contig yes map

group4 39650616 46287299 71 W ctg00472 1 6636684 -

group4 46287300 46287399 72 U 100 contig yes map

group4 46287400 47179397 73 W ctg00991 1 891998 -

group4 47179398 47179497 74 U 100 contig yes map

group4 47179498 48656594 75 W ctg00395 1 1477097 -

group4 48656595 48656694 76 U 100 contig yes map

group4 48656695 51021536 77 W ctg00288 1 2364842 -

group4 51021537 51021636 78 U 100 contig yes map

group4 51021637 52527628 79 W ctg00083 1 1505992 -

group4 52527629 52527728 80 U 100 contig yes map

group4 52527729 56419014 81 W ctg00811 1 3891286 -

group4 56419015 56419114 82 U 100 contig yes map

group4 56419115 56515991 83 W ctg00978 1 96877 +

group4 56515992 56516091 84 U 100 contig yes map

group4 56516092 56551631 85 W ctg00848 1 35540 -

group4 56551632 56551731 86 U 100 contig yes map

group4 56551732 56652334 87 W ctg00994 1 100603 +

group4 56652335 56652434 88 U 100 contig yes map

group4 56652435 58770022 89 W ctg00814 1 2117588 +

group4 58770023 58770122 90 U 100 contig yes map

group4 58770123 61576035 91 W ctg00207 1 2805913 -

group4 61576036 61576135 92 U 100 contig yes map

group4 61576136 62183978 93 W ctg00829 1 607843 -

group4 62183979 62184078 94 U 100 contig yes map

group4 62184079 63226695 95 W ctg00582 1 1042617 +

group4 63226696 63226795 96 U 100 contig yes map

group4 63226796 64222077 97 W ctg00690 1 995282 -

group4 64222078 64222177 98 U 100 contig yes map

group4 64222178 64410712 99 W ctg00586 1 188535 +

group4 64410713 64410812 100 U 100 contig yes map

group4 64410813 66904091 101 W ctg00828 1 2493279 -

group4 66904092 66904191 102 U 100 contig yes map

group4 66904192 68449411 103 W ctg00460 1 1545220 -

group4 68449412 68449511 104 U 100 contig yes map

group4 68449512 68492415 105 W ctg00899 1 42904 -

group4 68492416 68492515 106 U 100 contig yes map

group4 68492516 73630473 107 W ctg00170 1 5137958 +

group4 73630474 73630573 108 U 100 contig yes map

group4 73630574 73695723 109 W ctg00449 1 65150 -

group4 73695724 73695823 110 U 100 contig yes map

group4 73695824 73697559 111 W ctg00547 1 1736 +

group4 73697560 73697659 112 U 100 contig yes map

group4 73697660 75584261 113 W ctg00430 1 1886602 +

group4 75584262 75584361 114 U 100 contig yes map

group4 75584362 75609267 115 W ctg00501 1 24906 -

group4 75609268 75609367 116 U 100 contig yes map

group4 75609368 75627357 117 W ctg00275 1 17990 +

group4 75627358 75627457 118 U 100 contig yes map

group4 75627458 75695707 119 W ctg00376 1 68250 +

group4 75695708 75695807 120 U 100 contig yes map

group4 75695808 75746566 121 W ctg00566 1 50759 -

group4 75746567 75746666 122 U 100 contig yes map

group4 75746667 75798718 123 W ctg00263 1 52052 -

group4 75798719 75798818 124 U 100 contig yes map

group4 75798819 75856085 125 W ctg00762 1 57267 -

group4 75856086 75856185 126 U 100 contig yes map

group4 75856186 75915128 127 W ctg00500 1 58943 -

group4 75915129 75915228 128 U 100 contig yes map

group4 75915229 75953072 129 W ctg00703 1 37844 -

group4 75953073 75953172 130 U 100 contig yes map

group4 75953173 76117217 131 W ctg00550 1 164045 -

group4 76117218 76117317 132 U 100 contig yes map

group4 76117318 76156773 133 W ctg00359 1 39456 -

group4 76156774 76156873 134 U 100 contig yes map

group4 76156874 87570607 135 W ctg00880 1 11413734 +

group4 87570608 87570707 136 U 100 contig yes map

group4 87570708 87599375 137 W ctg00958 1 28668 -

group4 87599376 87599475 138 U 100 contig yes map

group4 87599476 90240149 139 W ctg00852 1 2640674 +

group4 90240150 90240249 140 U 100 contig yes map

group4 90240250 90307259 141 W ctg00592 1 67010 -

group4 90307260 90307359 142 U 100 contig yes map

group4 90307360 90370367 143 W ctg00134 1 63008 +

group4 90370368 90370467 144 U 100 contig yes map

group4 90370468 90519204 145 W ctg00101 1 148737 -

group4 90519205 90519304 146 U 100 contig yes map

group4 90519305 90610798 147 W ctg00746 1 91494 +

group4 90610799 90610898 148 U 100 contig yes map

group4 90610899 90691435 149 W ctg00689 1 80537 -

group4 90691436 90691535 150 U 100 contig yes map

group4 90691536 92583971 151 W ctg00454 1 1892436 +

group4 92583972 92584071 152 U 100 contig yes map

group4 92584072 100699676 153 W ctg00365 1 8115605 +

group4 100699677 100699776 154 U 100 contig yes map

group4 100699777 100710735 155 W ctg00817 1 10959 +

group4 100710736 100710835 156 U 100 contig yes map

group4 100710836 103985997 157 W ctg00716 1 3275162 -

group4 103985998 103986097 158 U 100 contig yes map

group4 103986098 104315147 159 W ctg00514 1 329050 +

group4 104315148 104315247 160 U 100 contig yes map

group4 104315248 104895231 161 W ctg01005 1 579984 -

group4 104895232 104895331 162 U 100 contig yes map

group4 104895332 106059493 163 W ctg00115 1 1164162 +

group4 106059494 106059593 164 U 100 contig yes map

group4 106059594 107519308 165 W ctg00882 1 1459715 -

group4 107519309 107519408 166 U 100 contig yes map

group4 107519409 108988902 167 W ctg00293 1 1469494 -

group4 108988903 108989002 168 U 100 contig yes map

group4 108989003 109181398 169 W ctg00805 1 192396 +

group4 109181399 109181498 170 U 100 contig yes map

group4 109181499 109207852 171 W ctg00650 1 26354 +

group4 109207853 109207952 172 U 100 contig yes map

group4 109207953 113403735 173 W ctg00846 1 4195783 -

group4 113403736 113403835 174 U 100 contig yes map

group4 113403836 113468696 175 W ctg00743 1 64861 +

group4 113468697 113468796 176 U 100 contig yes map

group4 113468797 113518694 177 W ctg00864 1 49898 -

group4 113518695 113518794 178 U 100 contig yes map

group4 113518795 113582497 179 W ctg00957 1 63703 -

group4 113582498 113582597 180 U 100 contig yes map

group4 113582598 113672993 181 W ctg00044 1 90396 -

group4 113672994 113673093 182 U 100 contig yes map

group4 113673094 113771313 183 W ctg00345 1 98220 +

group4 113771314 113771413 184 U 100 contig yes map

group4 113771414 113849844 185 W ctg00839 1 78431 +

group4 113849845 113849944 186 U 100 contig yes map

group4 113849945 114018286 187 W ctg00962 1 168342 -

group4 114018287 114018386 188 U 100 contig yes map

group4 114018387 115689516 189 W ctg00863 1 1671130 +

group4 115689517 115689616 190 U 100 contig yes map

group4 115689617 116894103 191 W ctg00404 1 1204487 +

group4 116894104 116894203 192 U 100 contig yes map

group4 116894204 117183496 193 W ctg00383 1 289293 -

group4 117183497 117183596 194 U 100 contig yes map

group4 117183597 117307395 195 W ctg00094 1 123799 -

group4 117307396 117307495 196 U 100 contig yes map

group4 117307496 117401932 197 W ctg00980 1 94437 -

group4 117401933 117402032 198 U 100 contig yes map

group4 117402033 120909763 199 W ctg00173 1 3507731 -

group4 120909764 120909863 200 U 100 contig yes map

group4 120909864 121224306 201 W ctg00085 1 314443 -

group4 121224307 121224406 202 U 100 contig yes map

group4 121224407 121339476 203 W ctg00687 1 115070 +

group4 121339477 121339576 204 U 100 contig yes map

group4 121339577 121543026 205 W ctg00054 1 203450 +

group4 121543027 121543126 206 U 100 contig yes map

group4 121543127 125379148 207 W ctg00515 1 3836022 -

group4 125379149 125379248 208 U 100 contig yes map

group4 125379249 125501534 209 W ctg00160 1 122286 +

group4 125501535 125501634 210 U 100 contig yes map

group4 125501635 127286968 211 W ctg00061 1 1785334 +

group4 127286969 127287068 212 U 100 contig yes map

group4 127287069 127930342 213 W ctg00437 1 643274 -

group4 127930343 127930442 214 U 100 contig yes map

group4 127930443 128093349 215 W ctg00933 1 162907 +

group4 128093350 128093449 216 U 100 contig yes map

group4 128093450 128547870 217 W ctg01006 1 454421 -

group4 128547871 128547970 218 U 100 contig yes map

group4 128547971 128621338 219 W ctg00911 1 73368 -

group4 128621339 128621438 220 U 100 contig yes map

group4 128621439 128820210 221 W ctg00198 1 198772 -

group4 128820211 128820310 222 U 100 contig yes map

group4 128820311 128900549 223 W ctg00429 1 80239 +

group4 128900550 128900649 224 U 100 contig yes map

group4 128900650 128979520 225 W ctg00069 1 78871 +

group4 128979521 128979620 226 U 100 contig yes map

group4 128979621 129030784 227 W ctg00090 1 51164 +

group4 129030785 129030884 228 U 100 contig yes map

group4 129030885 132636878 229 W ctg00673 1 3605994 +

group4 132636879 132636978 230 U 100 contig yes map

group4 132636979 132700067 231 W ctg00356 1 63089 -

group4 132700068 132700167 232 U 100 contig yes map

group4 132700168 132718714 233 W ctg00674 1 18547 -

group4 132718715 132718814 234 U 100 contig yes map

group4 132718815 132744663 235 W ctg00499 1 25849 -

group4 132744664 132744763 236 U 100 contig yes map

group4 132744764 132813536 237 W ctg00760 1 68773 -

group4 132813537 132813636 238 U 100 contig yes map

group4 132813637 132873403 239 W ctg00003 1 59767 -

group4 132873404 132873503 240 U 100 contig yes map

group4 132873504 132876161 241 W ctg00311 1 2658 -

group5 1 40196 1 W ctg00857 1 40196 +

group5 40197 40296 2 U 100 contig yes map

group5 40297 121993 3 W ctg00740 1 81697 +

group5 121994 122093 4 U 100 contig yes map

group5 122094 200838 5 W ctg00150 1 78745 +

group5 200839 200938 6 U 100 contig yes map

group5 200939 276945 7 W ctg00298 1 76007 +

group5 276946 277045 8 U 100 contig yes map

group5 277046 361830 9 W ctg00705 1 84785 -

group5 361831 361930 10 U 100 contig yes map

group5 361931 488605 11 W ctg00897 1 126675 -

group5 488606 488705 12 U 100 contig yes map

group5 488706 3303033 13 W ctg00366 1 2814328 +

group5 3303034 3303133 14 U 100 contig yes map

group5 3303134 3910066 15 W ctg00671 1 606933 +

group5 3910067 3910166 16 U 100 contig yes map

group5 3910167 4079765 17 W ctg00212 1 169599 +

group5 4079766 4079865 18 U 100 contig yes map

group5 4079866 6502598 19 W ctg00484 1 2422733 +

group5 6502599 6502698 20 U 100 contig yes map

group5 6502699 7131391 21 W ctg00091 1 628693 -

group5 7131392 7131491 22 U 100 contig yes map

group5 7131492 7298118 23 W ctg00752 1 166627 -

group5 7298119 7298218 24 U 100 contig yes map

group5 7298219 10026829 25 W ctg00505 1 2728611 +

group5 10026830 10026929 26 U 100 contig yes map

group5 10026930 13941884 27 W ctg00640 1 3914955 -

group5 13941885 13941984 28 U 100 contig yes map

group5 13941985 15753502 29 W ctg00942 1 1811518 -

group5 15753503 15753602 30 U 100 contig yes map

group5 15753603 15848242 31 W ctg00420 1 94640 +

group5 15848243 15848342 32 U 100 contig yes map

group5 15848343 15954644 33 W ctg00878 1 106302 +

group5 15954645 15954744 34 U 100 contig yes map

group5 15954745 21289246 35 W ctg00051 1 5334502 +

group5 21289247 21289346 36 U 100 contig yes map

group5 21289347 21389717 37 W ctg00046 1 100371 -

group5 21389718 21389817 38 U 100 contig yes map

group5 21389818 21450854 39 W ctg00218 1 61037 -

group5 21450855 21450954 40 U 100 contig yes map

group5 21450955 21539561 41 W ctg00021 1 88607 +

group5 21539562 21539661 42 U 100 contig yes map

group5 21539662 21609332 43 W ctg00633 1 69671 +

group5 21609333 21609432 44 U 100 contig yes map

group5 21609433 24738526 45 W ctg00237 1 3129094 -

group5 24738527 24738626 46 U 100 contig yes map

group5 24738627 24803246 47 W ctg00641 1 64620 -

group5 24803247 24803346 48 U 100 contig yes map

group5 24803347 32316871 49 W ctg00558 1 7513525 -

group5 32316872 32316971 50 U 100 contig yes map

group5 32316972 34584095 51 W ctg00210 1 2267124 -

group5 34584096 34584195 52 U 100 contig yes map

group5 34584196 34606708 53 W ctg00540 1 22513 -

group5 34606709 34606808 54 U 100 contig yes map

group5 34606809 35223716 55 W ctg00901 1 616908 -

group5 35223717 35223816 56 U 100 contig yes map

group5 35223817 35559398 57 W ctg00260 1 335582 -

group5 35559399 35559498 58 U 100 contig yes map

group5 35559499 44446660 59 W ctg00411 1 8887162 +

group5 44446661 44446760 60 U 100 contig yes map

group5 44446761 47528635 61 W ctg00277 1 3081875 +

group5 47528636 47528735 62 U 100 contig yes map

group5 47528736 57156930 63 W ctg00436 1 9628195 -

group5 57156931 57157030 64 U 100 contig yes map

group5 57157031 61489130 65 W ctg00358 1 4332100 -

group5 61489131 61489230 66 U 100 contig yes map

group5 61489231 61586337 67 W ctg00433 1 97107 +

group5 61586338 61586437 68 U 100 contig yes map

group5 61586438 61797725 69 W ctg00562 1 211288 -

group5 61797726 61797825 70 U 100 contig yes map

group5 61797826 61923696 71 W ctg00662 1 125871 -

group5 61923697 61923796 72 U 100 contig yes map

group5 61923797 74192395 73 W ctg00535 1 12268599 -

group5 74192396 74192495 74 U 100 contig yes map

group5 74192496 74230259 75 W ctg00721 1 37764 -

group5 74230260 74230359 76 U 100 contig yes map

group5 74230360 74293093 77 W ctg00342 1 62734 +

group5 74293094 74293193 78 U 100 contig yes map

group5 74293194 75662028 79 W ctg00983 1 1368835 +

group5 75662029 75662128 80 U 100 contig yes map

group5 75662129 80189437 81 W ctg00537 1 4527309 +

group5 80189438 80189537 82 U 100 contig yes map

group5 80189538 80290557 83 W ctg00448 1 101020 -

group5 80290558 80290657 84 U 100 contig yes map

group5 80290658 80426917 85 W ctg00610 1 136260 -

group5 80426918 80427017 86 U 100 contig yes map

group5 80427018 80511480 87 W ctg00738 1 84463 +

group5 80511481 80511580 88 U 100 contig yes map

group5 80511581 81594834 89 W ctg00385 1 1083254 -

group5 81594835 81594934 90 U 100 contig yes map

group5 81594935 81826075 91 W ctg00629 1 231141 +

group5 81826076 81826175 92 U 100 contig yes map

group5 81826176 81852174 93 W ctg00495 1 25999 -

group5 81852175 81852274 94 U 100 contig yes map

group5 81852275 82268817 95 W ctg00798 1 416543 +

group5 82268818 82268917 96 U 100 contig yes map

group5 82268918 85056699 97 W ctg00986 1 2787782 +

group5 85056700 85056799 98 U 100 contig yes map

group5 85056800 85915611 99 W ctg00121 1 858812 -

group5 85915612 85915711 100 U 100 contig yes map

group5 85915712 86643462 101 W ctg00140 1 727751 -

group5 86643463 86643562 102 U 100 contig yes map

group5 86643563 87933908 103 W ctg00400 1 1290346 -

group5 87933909 87934008 104 U 100 contig yes map

group5 87934009 88058044 105 W ctg00987 1 124036 +

group5 88058045 88058144 106 U 100 contig yes map

group5 88058145 100566767 107 W ctg00227 1 12508623 -

group5 100566768 100566867 108 U 100 contig yes map

group5 100566868 100584860 109 W ctg00779 1 17993 +

group5 100584861 100584960 110 U 100 contig yes map

group5 100584961 100618867 111 W ctg00766 1 33907 +

group5 100618868 100618967 112 U 100 contig yes map

group5 100618968 102593323 113 W ctg00329 1 1974356 -

group5 102593324 102593423 114 U 100 contig yes map

group5 102593424 104381020 115 W ctg00332 1 1787597 -

group5 104381021 104381120 116 U 100 contig yes map

group5 104381121 104427863 117 W ctg01023 1 46743 -

group5 104427864 104427963 118 U 100 contig yes map

group5 104427964 106625283 119 W ctg00089 1 2197320 -

group5 106625284 106625383 120 U 100 contig yes map

group5 106625384 112137093 121 W ctg00984 1 5511710 +

group5 112137094 112137193 122 U 100 contig yes map

group5 112137194 112224370 123 W ctg00564 1 87177 +

group5 112224371 112224470 124 U 100 contig yes map

group5 112224471 114244152 125 W ctg00462 1 2019682 +

group5 114244153 114244252 126 U 100 contig yes map

group5 114244253 115236050 127 W ctg00015 1 991798 -

group5 115236051 115236150 128 U 100 contig yes map

group5 115236151 117481551 129 W ctg00117 1 2245401 +

group5 117481552 117481651 130 U 100 contig yes map

group5 117481652 118748400 131 W ctg00686 1 1266749 +

group5 118748401 118748500 132 U 100 contig yes map

group5 118748501 120049642 133 W ctg00103 1 1301142 +

group5 120049643 120049742 134 U 100 contig yes map

group5 120049743 120134780 135 W ctg00244 1 85038 +

group5 120134781 120134880 136 U 100 contig yes map

group5 120134881 120217297 137 W ctg00578 1 82417 -

group5 120217298 120217397 138 U 100 contig yes map

group5 120217398 120235763 139 W ctg00214 1 18366 +

group5 120235764 120235863 140 U 100 contig yes map

group5 120235864 120266831 141 W ctg00380 1 30968 -

group5 120266832 120266931 142 U 100 contig yes map

group5 120266932 129207306 143 W ctg00708 1 8940375 +

group5 129207307 129207406 144 U 100 contig yes map

group5 129207407 131822954 145 W ctg00718 1 2615548 +

group5 131822955 131823054 146 U 100 contig yes map

group5 131823055 131824385 147 W ctg00424 1 1331 +

group5 131824386 131824485 148 U 100 contig yes map

group5 131824486 131826776 149 W ctg00951 1 2291 -

group5 131826777 131826876 150 U 100 contig yes map

group5 131826877 131906446 151 W ctg00555 1 79570 +

group6 1 229209 1 W ctg00912 1 229209 -

group6 229210 229309 2 U 100 contig yes map

group6 229310 419533 3 W ctg00097 1 190224 +

group6 419534 419633 4 U 100 contig yes map

group6 419634 446788 5 W ctg00145 1 27155 -

group6 446789 446888 6 U 100 contig yes map

group6 446889 680425 7 W ctg00137 1 233537 -

group6 680426 680525 8 U 100 contig yes map

group6 680526 4383640 9 W ctg00722 1 3703115 -

group6 4383641 4383740 10 U 100 contig yes map

group6 4383741 5273616 11 W ctg00129 1 889876 +

group6 5273617 5273716 12 U 100 contig yes map

group6 5273717 5673565 13 W ctg00491 1 399849 +

group6 5673566 5673665 14 U 100 contig yes map

group6 5673666 8899389 15 W ctg00026 1 3225724 +

group6 8899390 8899489 16 U 100 contig yes map

group6 8899490 12410969 17 W ctg00126 1 3511480 -

group6 12410970 12411069 18 U 100 contig yes map

group6 12411070 17162041 19 W ctg00967 1 4750972 +

group6 17162042 17162141 20 U 100 contig yes map

group6 17162142 17178834 21 W ctg00372 1 16693 +

group6 17178835 17178934 22 U 100 contig yes map

group6 17178935 17344664 23 W ctg00055 1 165730 +

group6 17344665 17344764 24 U 100 contig yes map

group6 17344765 18631099 25 W ctg00416 1 1286335 -

group6 18631100 18631199 26 U 100 contig yes map

group6 18631200 24800377 27 W ctg00270 1 6169178 -

group6 24800378 24800477 28 U 100 contig yes map

group6 24800478 28067896 29 W ctg00073 1 3267419 -

group6 28067897 28067996 30 U 100 contig yes map

group6 28067997 29052581 31 W ctg00845 1 984585 -

group6 29052582 29052681 32 U 100 contig yes map

group6 29052682 31634551 33 W ctg00583 1 2581870 -

group6 31634552 31634651 34 U 100 contig yes map

group6 31634652 36647957 35 W ctg00679 1 5013306 +

group6 36647958 36648057 36 U 100 contig yes map

group6 36648058 38682260 37 W ctg00139 1 2034203 +

group6 38682261 38682360 38 U 100 contig yes map

group6 38682361 38820458 39 W ctg00231 1 138098 -

group6 38820459 38820558 40 U 100 contig yes map

group6 38820559 39253458 41 W ctg00770 1 432900 -

group6 39253459 39253558 42 U 100 contig yes map

group6 39253559 42778292 43 W ctg00067 1 3524734 -

group6 42778293 42778392 44 U 100 contig yes map

group6 42778393 43442274 45 W ctg00658 1 663882 +

group6 43442275 43442374 46 U 100 contig yes map

group6 43442375 44135603 47 W ctg00590 1 693229 -

group6 44135604 44135703 48 U 100 contig yes map

group6 44135704 44706139 49 W ctg00885 1 570436 +

group6 44706140 44706239 50 U 100 contig yes map

group6 44706240 50216199 51 W ctg00378 1 5509960 -

group6 50216200 50216299 52 U 100 contig yes map

group6 50216300 54388691 53 W ctg00685 1 4172392 -

group6 54388692 54388791 54 U 100 contig yes map

group6 54388792 66600378 55 W ctg00683 1 12211587 +

group6 66600379 66600478 56 U 100 contig yes map

group6 66600479 68117614 57 W ctg00646 1 1517136 -

group6 68117615 68117714 58 U 100 contig yes map

group6 68117715 68677132 59 W ctg00191 1 559418 -

group6 68677133 68677232 60 U 100 contig yes map

group6 68677233 68763939 61 W ctg00631 1 86707 +

group6 68763940 68764039 62 U 100 contig yes map

group6 68764040 73666052 63 W ctg00337 1 4902013 +

group6 73666053 73666152 64 U 100 contig yes map

group6 73666153 80935267 65 W ctg00968 1 7269115 -

group6 80935268 80935367 66 U 100 contig yes map

group6 80935368 81002962 67 W ctg00407 1 67595 -

group6 81002963 81003062 68 U 100 contig yes map

group6 81003063 82071802 69 W ctg00919 1 1068740 -

group6 82071803 82071902 70 U 100 contig yes map

group6 82071903 87620338 71 W ctg00068 1 5548436 -

group6 87620339 87620438 72 U 100 contig yes map

group6 87620439 92833389 73 W ctg00796 1 5212951 -

group6 92833390 92833489 74 U 100 contig yes map

group6 92833490 94045815 75 W ctg00119 1 1212326 -

group6 94045816 94045915 76 U 100 contig yes map

group6 94045916 94141674 77 W ctg00832 1 95759 +

group6 94141675 94141774 78 U 100 contig yes map

group6 94141775 94205885 79 W ctg00338 1 64111 +

group6 94205886 94205985 80 U 100 contig yes map

group6 94205986 105733342 81 W ctg00997 1 11527357 -

group6 105733343 105733442 82 U 100 contig yes map

group6 105733443 105798325 83 W ctg01004 1 64883 -

group6 105798326 105798425 84 U 100 contig yes map

group6 105798426 105823714 85 W ctg00136 1 25289 +

group6 105823715 105823814 86 U 100 contig yes map

group6 105823815 106817276 87 W ctg00445 1 993462 -

group6 106817277 106817376 88 U 100 contig yes map

group6 106817377 106875624 89 W ctg00541 1 58248 +

group6 106875625 106875724 90 U 100 contig yes map

group6 106875725 107277166 91 W ctg00636 1 401442 -

group6 107277167 107277266 92 U 100 contig yes map

group6 107277267 107507768 93 W ctg00116 1 230502 -

group6 107507769 107507868 94 U 100 contig yes map

group6 107507869 107535348 95 W ctg00428 1 27480 +

group6 107535349 107535448 96 U 100 contig yes map

group6 107535449 107759504 97 W ctg00733 1 224056 +

group6 107759505 107759604 98 U 100 contig yes map

group6 107759605 107939849 99 W ctg00533 1 180245 +

group6 107939850 107939949 100 U 100 contig yes map

group6 107939950 108032596 101 W ctg00406 1 92647 +

group6 108032597 108032696 102 U 100 contig yes map

group6 108032697 117559005 103 W ctg00075 1 9526309 +

group6 117559006 117559105 104 U 100 contig yes map

group6 117559106 117644885 105 W ctg00243 1 85780 -

group6 117644886 117644985 106 U 100 contig yes map

group6 117644986 117765790 107 W ctg00112 1 120805 -

group6 117765791 117765890 108 U 100 contig yes map

group6 117765891 118284295 109 W ctg00714 1 518405 +

group6 118284296 118284395 110 U 100 contig yes map

group6 118284396 122156715 111 W ctg00289 1 3872320 +

group6 122156716 122156815 112 U 100 contig yes map

group6 122156816 122183558 113 W ctg00084 1 26743 +

group6 122183559 122183658 114 U 100 contig yes map

group6 122183659 122919650 115 W ctg00937 1 735992 +

group6 122919651 122919750 116 U 100 contig yes map

group6 122919751 124043458 117 W ctg00630 1 1123708 +

group6 124043459 124043558 118 U 100 contig yes map

group6 124043559 124119229 119 W ctg00470 1 75671 -

group6 124119230 124119329 120 U 100 contig yes map

group6 124119330 125772712 121 W ctg00381 1 1653383 -

group6 125772713 125772812 122 U 100 contig yes map

group6 125772813 126108534 123 W ctg00735 1 335722 +

group6 126108535 126108634 124 U 100 contig yes map

group6 126108635 126269452 125 W ctg00325 1 160818 +

group6 126269453 126269552 126 U 100 contig yes map

group6 126269553 126363402 127 W ctg00326 1 93850 +

group6 126363403 126363502 128 U 100 contig yes map

group6 126363503 126413597 129 W ctg00379 1 50095 -

group6 126413598 126413697 130 U 100 contig yes map

group6 126413698 126453657 131 W ctg00737 1 39960 +

group6 126453658 126453757 132 U 100 contig yes map

group6 126453758 126532522 133 W ctg00006 1 78765 -

group6 126532523 126532622 134 U 100 contig yes map

group6 126532623 126577075 135 W ctg00523 1 44453 -

group6 126577076 126577175 136 U 100 contig yes map

group6 126577176 126612398 137 W ctg00643 1 35223 +

group6 126612399 126612498 138 U 100 contig yes map

group6 126612499 126670429 139 W ctg00860 1 57931 -

group6 126670430 126670529 140 U 100 contig yes map

group6 126670530 126735801 141 W ctg00836 1 65272 +

group6 126735802 126735901 142 U 100 contig yes map

group6 126735902 126786868 143 W ctg00197 1 50967 +

group6 126786869 126786968 144 U 100 contig yes map

group6 126786969 126834507 145 W ctg00104 1 47539 +

group6 126834508 126834607 146 U 100 contig yes map

group6 126834608 126902673 147 W ctg00242 1 68066 -

group6 126902674 126902773 148 U 100 contig yes map

group6 126902774 126961814 149 W ctg00219 1 59041 -

group6 126961815 126961914 150 U 100 contig yes map

group6 126961915 127072541 151 W ctg00915 1 110627 -

group6 127072542 127072641 152 U 100 contig yes map

group6 127072642 127128519 153 W ctg00570 1 55878 +

group6 127128520 127128619 154 U 100 contig yes map

group6 127128620 127177211 155 W ctg00621 1 48592 -

group6 127177212 127177311 156 U 100 contig yes map

group6 127177312 127308935 157 W ctg00152 1 131624 -

group6 127308936 127309035 158 U 100 contig yes map

group6 127309036 127366112 159 W ctg00808 1 57077 +

group6 127366113 127366212 160 U 100 contig yes map

group6 127366213 127428598 161 W ctg00459 1 62386 +

group6 127428599 127428698 162 U 100 contig yes map

group6 127428699 127504546 163 W ctg00295 1 75848 +

group6 127504547 127504646 164 U 100 contig yes map

group6 127504647 127627235 165 W ctg00799 1 122589 -

group6 127627236 127627335 166 U 100 contig yes map

group6 127627336 127685993 167 W ctg00668 1 58658 -

group6 127685994 127686093 168 U 100 contig yes map

group6 127686094 127746217 169 W ctg00961 1 60124 -

group6 127746218 127746317 170 U 100 contig yes map

group6 127746318 127851921 171 W ctg00974 1 105604 +

group6 127851922 127852021 172 U 100 contig yes map

group6 127852022 127926725 173 W ctg00959 1 74704 +

group6 127926726 127926825 174 U 100 contig yes map

group6 127926826 127989202 175 W ctg00728 1 62377 +

group6 127989203 127989302 176 U 100 contig yes map

group6 127989303 128047712 177 W ctg00413 1 58410 +

group6 128047713 128047812 178 U 100 contig yes map

group6 128047813 128182000 179 W ctg00245 1 134188 -

group6 128182001 128182100 180 U 100 contig yes map

group6 128182101 128249250 181 W ctg00929 1 67150 +

group6 128249251 128249350 182 U 100 contig yes map

group6 128249351 128305696 183 W ctg00473 1 56346 +

group6 128305697 128305796 184 U 100 contig yes map

group6 128305797 128332931 185 W ctg00391 1 27135 +

group6 128332932 128333031 186 U 100 contig yes map

group6 128333032 128377589 187 W ctg00393 1 44558 +

group6 128377590 128377689 188 U 100 contig yes map

group6 128377690 128431475 189 W ctg00952 1 53786 -

group6 128431476 128431575 190 U 100 contig yes map

group6 128431576 128480701 191 W ctg00032 1 49126 +

group6 128480702 128480801 192 U 100 contig yes map

group6 128480802 128520500 193 W ctg00659 1 39699 -

group6 128520501 128520600 194 U 100 contig yes map

group6 128520601 128582059 195 W ctg00502 1 61459 -

group6 128582060 128582159 196 U 100 contig yes map

group6 128582160 128636821 197 W ctg00486 1 54662 -

group6 128636822 128636921 198 U 100 contig yes map

group6 128636922 128710402 199 W ctg00398 1 73481 -

group6 128710403 128710502 200 U 100 contig yes map

group6 128710503 128769032 201 W ctg00830 1 58530 -

group6 128769033 128769132 202 U 100 contig yes map

group6 128769133 128828797 203 W ctg00910 1 59665 +

group6 128828798 128828897 204 U 100 contig yes map

group6 128828898 128907231 205 W ctg00813 1 78334 +

group6 128907232 128907331 206 U 100 contig yes map

group6 128907332 128975249 207 W ctg00327 1 67918 +

group6 128975250 128975349 208 U 100 contig yes map

group6 128975350 129020182 209 W ctg00538 1 44833 -

group6 129020183 129020282 210 U 100 contig yes map

group6 129020283 129064387 211 W ctg00542 1 44105 -

group6 129064388 129064487 212 U 100 contig yes map

group6 129064488 129137004 213 W ctg00399 1 72517 -

group6 129137005 129137104 214 U 100 contig yes map

group6 129137105 129186366 215 W ctg00819 1 49262 +

group6 129186367 129186466 216 U 100 contig yes map

group6 129186467 129223753 217 W ctg00767 1 37287 +

group6 129223754 129223853 218 U 100 contig yes map

group6 129223854 129325394 219 W ctg00763 1 101541 -

group6 129325395 129325494 220 U 100 contig yes map

group6 129325495 129364720 221 W ctg00354 1 39226 +

group6 129364721 129364820 222 U 100 contig yes map

group6 129364821 129397736 223 W ctg00109 1 32916 -

group6 129397737 129397836 224 U 100 contig yes map

group6 129397837 129453629 225 W ctg00193 1 55793 -

group6 129453630 129453729 226 U 100 contig yes map

group6 129453730 129486450 227 W ctg00011 1 32721 +

group6 129486451 129486550 228 U 100 contig yes map

group6 129486551 129525345 229 W ctg00614 1 38795 +

group6 129525346 129525445 230 U 100 contig yes map

group6 129525446 129567132 231 W ctg00516 1 41687 +

group6 129567133 129567232 232 U 100 contig yes map

group6 129567233 129609413 233 W ctg00534 1 42181 +

group6 129609414 129609513 234 U 100 contig yes map

group6 129609514 129673303 235 W ctg01002 1 63790 +

group6 129673304 129673403 236 U 100 contig yes map

group6 129673404 129762216 237 W ctg00132 1 88813 -

group6 129762217 129762316 238 U 100 contig yes map

group6 129762317 129935116 239 W ctg00664 1 172800 +

group6 129935117 129935216 240 U 100 contig yes map

group6 129935217 130007377 241 W ctg00665 1 72161 +

group6 130007378 130007477 242 U 100 contig yes map

group6 130007478 130057214 243 W ctg00388 1 49737 -

group6 130057215 130057314 244 U 100 contig yes map

group6 130057315 130097656 245 W ctg00363 1 40342 +

group6 130097657 130097756 246 U 100 contig yes map

group6 130097757 130167193 247 W ctg00113 1 69437 +

group6 130167194 130167293 248 U 100 contig yes map

group6 130167294 130279067 249 W ctg00415 1 111774 -

group6 130279068 130279167 250 U 100 contig yes map

group6 130279168 130351009 251 W ctg00229 1 71842 -

group6 130351010 130351109 252 U 100 contig yes map

group6 130351110 130413139 253 W ctg00334 1 62030 +

group6 130413140 130413239 254 U 100 contig yes map

group6 130413240 130455027 255 W ctg00466 1 41788 +

group6 130455028 130455127 256 U 100 contig yes map

group6 130455128 130489488 257 W ctg00464 1 34361 -

group6 130489489 130489588 258 U 100 contig yes map

group6 130489589 130542091 259 W ctg00221 1 52503 +

group6 130542092 130542191 260 U 100 contig yes map

group6 130542192 130605428 261 W ctg00066 1 63237 +

group6 130605429 130605528 262 U 100 contig yes map

group6 130605529 130730051 263 W ctg00758 1 124523 -

group6 130730052 130730151 264 U 100 contig yes map

group6 130730152 130777329 265 W ctg00551 1 47178 -

group6 130777330 130777429 266 U 100 contig yes map

group6 130777430 130853512 267 W ctg00076 1 76083 -

group6 130853513 130853612 268 U 100 contig yes map

group6 130853613 130930662 269 W ctg00184 1 77050 +

group6 130930663 130930762 270 U 100 contig yes map

group6 130930763 130962998 271 W ctg00082 1 32236 +

group6 130962999 130963098 272 U 100 contig yes map

group6 130963099 131039865 273 W ctg00007 1 76767 +

group6 131039866 131039965 274 U 100 contig yes map

group6 131039966 131102570 275 W ctg00626 1 62605 +

group6 131102571 131102670 276 U 100 contig yes map

group6 131102671 131152061 277 W ctg00694 1 49391 -

group6 131152062 131152161 278 U 100 contig yes map

group6 131152162 131205527 279 W ctg01007 1 53366 -

group6 131205528 131205627 280 U 100 contig yes map

group6 131205628 131262842 281 W ctg00954 1 57215 +

group6 131262843 131262942 282 U 100 contig yes map

group6 131262943 131320411 283 W ctg00320 1 57469 -

group6 131320412 131320511 284 U 100 contig yes map

group6 131320512 131369469 285 W ctg00909 1 48958 -

group6 131369470 131369569 286 U 100 contig yes map

group6 131369570 131410087 287 W ctg01013 1 40518 -

group6 131410088 131410187 288 U 100 contig yes map

group6 131410188 131470249 289 W ctg00223 1 60062 +

group6 131470250 131470349 290 U 100 contig yes map

group6 131470350 131519915 291 W ctg00488 1 49566 +

group6 131519916 131520015 292 U 100 contig yes map

group6 131520016 131598890 293 W ctg00149 1 78875 -

group6 131598891 131598990 294 U 100 contig yes map

group6 131598991 131633123 295 W ctg00862 1 34133 +

group6 131633124 131633223 296 U 100 contig yes map

group6 131633224 131691151 297 W ctg00247 1 57928 -

group6 131691152 131691251 298 U 100 contig yes map

group6 131691252 131739076 299 W ctg00201 1 47825 +

group6 131739077 131739176 300 U 100 contig yes map

group6 131739177 131792828 301 W ctg00904 1 53652 +

group6 131792829 131792928 302 U 100 contig yes map

group6 131792929 131820655 303 W ctg00419 1 27727 -

group6 131820656 131820755 304 U 100 contig yes map

group6 131820756 131862183 305 W ctg00278 1 41428 +

group6 131862184 131862283 306 U 100 contig yes map

group6 131862284 131911048 307 W ctg00637 1 48765 +

group6 131911049 131911148 308 U 100 contig yes map

group6 131911149 131947187 309 W ctg01026 1 36039 -

group6 131947188 131947287 310 U 100 contig yes map

group6 131947288 131989454 311 W ctg00519 1 42167 +

group6 131989455 131989554 312 U 100 contig yes map

group6 131989555 132009641 313 W ctg00330 1 20087 +

group6 132009642 132009741 314 U 100 contig yes map

group6 132009742 132071790 315 W ctg00125 1 62049 -

group6 132071791 132071890 316 U 100 contig yes map

group6 132071891 132127159 317 W ctg00657 1 55269 -

group6 132127160 132127259 318 U 100 contig yes map

group6 132127260 132163635 319 W ctg00771 1 36376 -

group6 132163636 132163735 320 U 100 contig yes map

group6 132163736 132222668 321 W ctg00042 1 58933 +

group6 132222669 132222768 322 U 100 contig yes map

group6 132222769 132271129 323 W ctg00016 1 48361 +

group6 132271130 132271229 324 U 100 contig yes map

group6 132271230 132310972 325 W ctg00568 1 39743 -

group6 132310973 132311072 326 U 100 contig yes map

group6 132311073 132350123 327 W ctg00532 1 39051 +

group6 132350124 132350223 328 U 100 contig yes map

group6 132350224 132467089 329 W ctg00943 1 116866 -

group6 132467090 132467189 330 U 100 contig yes map

group6 132467190 132652155 331 W ctg00782 1 184966 -

group6 132652156 132652255 332 U 100 contig yes map

group6 132652256 132713545 333 W ctg00291 1 61290 -

group6 132713546 132713645 334 U 100 contig yes map

group6 132713646 132762726 335 W ctg00317 1 49081 -

group6 132762727 132762826 336 U 100 contig yes map

group6 132762827 132825270 337 W ctg00328 1 62444 -

group6 132825271 132825370 338 U 100 contig yes map

group6 132825371 132855752 339 W ctg00434 1 30382 +

group6 132855753 132855852 340 U 100 contig yes map

group6 132855853 132885056 341 W ctg00525 1 29204 -

group6 132885057 132885156 342 U 100 contig yes map

group6 132885157 132912355 343 W ctg00333 1 27199 -

group6 132912356 132912455 344 U 100 contig yes map

group6 132912456 132963947 345 W ctg00343 1 51492 -

group6 132963948 132964047 346 U 100 contig yes map

group6 132964048 133011807 347 W ctg00924 1 47760 -

group6 133011808 133011907 348 U 100 contig yes map

group6 133011908 133015006 349 W ctg00715 1 3099 -

group7 1 85901 1 W ctg00601 1 85901 -

group7 85902 86001 2 U 100 contig yes map

group7 86002 119898 3 W ctg01009 1 33897 -

group7 119899 119998 4 U 100 contig yes map

group7 119999 7951865 5 W ctg00598 1 7831867 -

group7 7951866 7951965 6 U 100 contig yes map

group7 7951966 7996872 7 W ctg00204 1 44907 -

group7 7996873 7996972 8 U 100 contig yes map

group7 7996973 11913916 9 W ctg00480 1 3916944 -

group7 11913917 11914016 10 U 100 contig yes map

group7 11914017 13453299 11 W ctg00574 1 1539283 +

group7 13453300 13453399 12 U 100 contig yes map

group7 13453400 13554386 13 W ctg00431 1 100987 -

group7 13554387 13554486 14 U 100 contig yes map

group7 13554487 13576397 15 W ctg00294 1 21911 +

group7 13576398 13576497 16 U 100 contig yes map

group7 13576498 13726825 17 W ctg01012 1 150328 +

group7 13726826 13726925 18 U 100 contig yes map

group7 13726926 16527659 19 W ctg00230 1 2800734 +

group7 16527660 16527759 20 U 100 contig yes map

group7 16527760 16639297 21 W ctg01014 1 111538 -

group7 16639298 16639397 22 U 100 contig yes map

group7 16639398 21331155 23 W ctg00754 1 4691758 -

group7 21331156 21331255 24 U 100 contig yes map

group7 21331256 29820763 25 W ctg01022 1 8489508 -

group7 29820764 29820863 26 U 100 contig yes map

group7 29820864 30275232 27 W ctg00382 1 454369 -

group7 30275233 30275332 28 U 100 contig yes map

group7 30275333 40741777 29 W ctg00648 1 10466445 -

group7 40741778 40741877 30 U 100 contig yes map

group7 40741878 40821121 31 W ctg00019 1 79244 -

group7 40821122 40821221 32 U 100 contig yes map

group7 40821222 41182755 33 W ctg00651 1 361534 +

group7 41182756 41182855 34 U 100 contig yes map

group7 41182856 41451248 35 W ctg00181 1 268393 +

group7 41451249 41451348 36 U 100 contig yes map

group7 41451349 41495542 37 W ctg00035 1 44194 -

group7 41495543 41495642 38 U 100 contig yes map

group7 41495643 41551612 39 W ctg00736 1 55970 -

group7 41551613 41551712 40 U 100 contig yes map

group7 41551713 42512568 41 W ctg00971 1 960856 -

group7 42512569 42512668 42 U 100 contig yes map

group7 42512669 45725407 43 W ctg00014 1 3212739 +

group7 45725408 45725507 44 U 100 contig yes map

group7 45725508 45774134 45 W ctg00301 1 48627 -

group7 45774135 45774234 46 U 100 contig yes map

group7 45774235 45826635 47 W ctg00169 1 52401 -

group7 45826636 45826735 48 U 100 contig yes map

group7 45826736 45947700 49 W ctg00336 1 120965 -

group7 45947701 45947800 50 U 100 contig yes map

group7 45947801 46000538 51 W ctg00700 1 52738 +

group7 46000539 46000638 52 U 100 contig yes map

group7 46000639 46122141 53 W ctg00589 1 121503 -

group7 46122142 46122241 54 U 100 contig yes map

group7 46122242 46375995 55 W ctg00079 1 253754 +

group7 46375996 46376095 56 U 100 contig yes map

group7 46376096 46419804 57 W ctg00888 1 43709 -

group7 46419805 46419904 58 U 100 contig yes map

group7 46419905 46532513 59 W ctg00318 1 112609 -

group7 46532514 46532613 60 U 100 contig yes map

group7 46532614 46600026 61 W ctg00421 1 67413 +

group7 46600027 46600126 62 U 100 contig yes map

group7 46600127 48574364 63 W ctg00955 1 1974238 -

group7 48574365 48574464 64 U 100 contig yes map

group7 48574465 51414008 65 W ctg00667 1 2839544 -

group7 51414009 51414108 66 U 100 contig yes map

group7 51414109 52938481 67 W ctg00508 1 1524373 -

group7 52938482 52938581 68 U 100 contig yes map

group7 52938582 54369086 69 W ctg00544 1 1430505 +

group7 54369087 54369186 70 U 100 contig yes map

group7 54369187 55103364 71 W ctg00692 1 734178 +

group7 55103365 55103464 72 U 100 contig yes map

group7 55103465 55158379 73 W ctg00171 1 54915 +

group7 55158380 55158479 74 U 100 contig yes map

group7 55158480 57915327 75 W ctg00834 1 2756848 -

group7 57915328 57915427 76 U 100 contig yes map

group7 57915428 60081349 77 W ctg00941 1 2165922 -

group7 60081350 60081449 78 U 100 contig yes map

group7 60081450 60146190 79 W ctg00340 1 64741 +

group7 60146191 60146290 80 U 100 contig yes map

group7 60146291 60233152 81 W ctg00596 1 86862 -

group7 60233153 60233252 82 U 100 contig yes map

group7 60233253 62824679 83 W ctg00147 1 2591427 +

group7 62824680 62824779 84 U 100 contig yes map

group7 62824780 62894698 85 W ctg00036 1 69919 +

group7 62894699 62894798 86 U 100 contig yes map

group7 62894799 67282518 87 W ctg00192 1 4387720 +

group7 67282519 67282618 88 U 100 contig yes map

group7 67282619 67366437 89 W ctg00373 1 83819 +

group7 67366438 67366537 90 U 100 contig yes map

group7 67366538 69513659 91 W ctg00236 1 2147122 -

group7 69513660 69513759 92 U 100 contig yes map

group7 69513760 75760039 93 W ctg00034 1 6246280 -

group7 75760040 75760139 94 U 100 contig yes map

group7 75760140 75817115 95 W ctg00838 1 56976 -

group7 75817116 75817215 96 U 100 contig yes map

group7 75817216 75903755 97 W ctg00029 1 86540 +

group7 75903756 75903855 98 U 100 contig yes map

group7 75903856 78598676 99 W ctg00476 1 2694821 -

group7 78598677 78598776 100 U 100 contig yes map

group7 78598777 79990955 101 W ctg00652 1 1392179 +

group7 79990956 79991055 102 U 100 contig yes map

group7 79991056 86388172 103 W ctg00106 1 6397117 +

group7 86388173 86388272 104 U 100 contig yes map

group7 86388273 90023140 105 W ctg00528 1 3634868 +

group7 90023141 90023240 106 U 100 contig yes map

group7 90023241 92942039 107 W ctg00020 1 2918799 +

group7 92942040 92942139 108 U 100 contig yes map

group7 92942140 97773329 109 W ctg00588 1 4831190 -

group7 97773330 97773429 110 U 100 contig yes map

group7 97773430 97831927 111 W ctg00923 1 58498 -

group7 97831928 97832027 112 U 100 contig yes map

group7 97832028 100220290 113 W ctg00985 1 2388263 -

group7 100220291 100220390 114 U 100 contig yes map

group7 100220391 100707361 115 W ctg00444 1 486971 +

group7 100707362 100707461 116 U 100 contig yes map

group7 100707462 109160824 117 W ctg00158 1 8453363 -

group7 109160825 109160924 118 U 100 contig yes map

group7 109160925 109242029 119 W ctg00747 1 81105 -

group7 109242030 109242129 120 U 100 contig yes map

group7 109242130 109403378 121 W ctg01017 1 161249 -

group7 109403379 109403478 122 U 100 contig yes map

group7 109403479 109503607 123 W ctg00953 1 100129 -

group7 109503608 109503707 124 U 100 contig yes map

group7 109503708 109621607 125 W ctg00412 1 117900 -

group7 109621608 109621707 126 U 100 contig yes map

group7 109621708 111564806 127 W ctg00315 1 1943099 -

group7 111564807 111564906 128 U 100 contig yes map

group7 111564907 112190358 129 W ctg00396 1 625452 +

group7 112190359 112190458 130 U 100 contig yes map

group7 112190459 112810200 131 W ctg00200 1 619742 +

group7 112810201 112810300 132 U 100 contig yes map

group7 112810301 112913296 133 W ctg00240 1 102996 +

group7 112913297 112913396 134 U 100 contig yes map

group7 112913397 113192302 135 W ctg00423 1 278906 -

group7 113192303 113192402 136 U 100 contig yes map

group7 113192403 113301488 137 W ctg00481 1 109086 -

group7 113301489 113301588 138 U 100 contig yes map

group7 113301589 115958946 139 W ctg00932 1 2657358 +

group7 115958947 115959046 140 U 100 contig yes map

group7 115959047 116109705 141 W ctg00884 1 150659 -

group7 116109706 116109805 142 U 100 contig yes map

group7 116109806 116439454 143 W ctg00199 1 329649 -

group7 116439455 116439554 144 U 100 contig yes map

group7 116439555 116765846 145 W ctg00711 1 326292 +

group7 116765847 116765946 146 U 100 contig yes map

group7 116765947 117753215 147 W ctg00989 1 987269 +

group8 1 2703 1 W ctg00286 1 2703 -

group8 2704 2803 2 U 100 contig yes map

group8 2804 77702 3 W ctg00252 1 74899 +

group8 77703 77802 4 U 100 contig yes map

group8 77803 148532 5 W ctg00108 1 70730 -

group8 148533 148632 6 U 100 contig yes map

group8 148633 219872 7 W ctg00926 1 71240 -

group8 219873 219972 8 U 100 contig yes map

group8 219973 270002 9 W ctg00693 1 50030 +

group8 270003 270102 10 U 100 contig yes map

group8 270103 272365 11 W ctg00918 1 2263 -

group8 272366 272465 12 U 100 contig yes map

group8 272466 519670 13 W ctg00257 1 247205 +

group8 519671 519770 14 U 100 contig yes map

group8 519771 1031461 15 W ctg00127 1 511691 -

group8 1031462 1031561 16 U 100 contig yes map

group8 1031562 1723508 17 W ctg00496 1 691947 +

group8 1723509 1723608 18 U 100 contig yes map

group8 1723609 3641657 19 W ctg00778 1 1918049 +

group8 3641658 3641757 20 U 100 contig yes map

group8 3641758 3724597 21 W ctg00172 1 82840 -

group8 3724598 3724697 22 U 100 contig yes map

group8 3724698 3800472 23 W ctg00238 1 75775 -

group8 3800473 3800572 24 U 100 contig yes map

group8 3800573 10739777 25 W ctg00719 1 6939205 -

group8 10739778 10739877 26 U 100 contig yes map

group8 10739878 10744137 27 W ctg00604 1 4260 -

group8 10744138 10744237 28 U 100 contig yes map

group8 10744238 10816546 29 W ctg00764 1 72309 +

group8 10816547 10816646 30 U 100 contig yes map

group8 10816647 10861613 31 W ctg00074 1 44967 -

group8 10861614 10861713 32 U 100 contig yes map

group8 10861714 11421744 33 W ctg00233 1 560031 -

group8 11421745 11421844 34 U 100 contig yes map

group8 11421845 12954588 35 W ctg00050 1 1532744 +

group8 12954589 12954688 36 U 100 contig yes map

group8 12954689 12965690 37 W ctg00259 1 11002 -

group8 12965691 12965790 38 U 100 contig yes map

group8 12965791 14072690 39 W ctg00654 1 1106900 +

group8 14072691 14072790 40 U 100 contig yes map

group8 14072791 14148100 41 W ctg00018 1 75310 -

group8 14148101 14148200 42 U 100 contig yes map

group8 14148201 14216441 43 W ctg00049 1 68241 -

group8 14216442 14216541 44 U 100 contig yes map

group8 14216542 20044985 45 W ctg00510 1 5828444 -

group8 20044986 20045085 46 U 100 contig yes map

group8 20045086 20106030 47 W ctg00585 1 60945 -

group8 20106031 20106130 48 U 100 contig yes map

group8 20106131 20358278 49 W ctg00831 1 252148 +

group8 20358279 20358378 50 U 100 contig yes map

group8 20358379 27906669 51 W ctg00887 1 7548291 -

group8 27906670 27906769 52 U 100 contig yes map

group8 27906770 30901217 53 W ctg00344 1 2994448 -

group8 30901218 30901317 54 U 100 contig yes map

group8 30901318 31004956 55 W ctg00310 1 103639 +

group8 31004957 31005056 56 U 100 contig yes map

group8 31005057 31235259 57 W ctg00447 1 230203 +

group8 31235260 31235359 58 U 100 contig yes map

group8 31235360 33961699 59 W ctg00048 1 2726340 +

group8 33961700 33961799 60 U 100 contig yes map

group8 33961800 35802919 61 W ctg00579 1 1841120 -

group8 35802920 35803019 62 U 100 contig yes map

group8 35803020 35811980 63 W ctg00308 1 8961 +

group8 35811981 35812080 64 U 100 contig yes map

group8 35812081 35862228 65 W ctg00306 1 50148 -

group8 35862229 35862328 66 U 100 contig yes map

group8 35862329 36009732 67 W ctg00410 1 147404 +

group8 36009733 36009832 68 U 100 contig yes map

group8 36009833 36052696 69 W ctg00725 1 42864 -

group8 36052697 36052796 70 U 100 contig yes map

group8 36052797 36160532 71 W ctg00999 1 107736 -

group8 36160533 36160632 72 U 100 contig yes map

group8 36160633 36278272 73 W ctg00591 1 117640 -

group8 36278273 36278372 74 U 100 contig yes map

group8 36278373 36760248 75 W ctg00938 1 481876 -

group8 36760249 36760348 76 U 100 contig yes map

group8 36760349 37834906 77 W ctg00065 1 1074558 +

group8 37834907 37835006 78 U 100 contig yes map

group8 37835007 37919028 79 W ctg00886 1 84022 +

group8 37919029 37919128 80 U 100 contig yes map

group8 37919129 38142260 81 W ctg00450 1 223132 -

group8 38142261 38142360 82 U 100 contig yes map

group8 38142361 38207668 83 W ctg00556 1 65308 +

group8 38207669 38207768 84 U 100 contig yes map

group8 38207769 38236338 85 W ctg00493 1 28570 +

group8 38236339 38236438 86 U 100 contig yes map

group8 38236439 38326651 87 W ctg00375 1 90213 -

group8 38326652 38326751 88 U 100 contig yes map

group8 38326752 38474179 89 W ctg00348 1 147428 -

group8 38474180 38474279 90 U 100 contig yes map

group8 38474280 39442859 91 W ctg00138 1 968580 +

group8 39442860 39442959 92 U 100 contig yes map

group8 39442960 39475882 93 W ctg00569 1 32923 -

group8 39475883 39475982 94 U 100 contig yes map

group8 39475983 40577445 95 W ctg00945 1 1101463 -

group8 40577446 40577545 96 U 100 contig yes map

group8 40577546 44389379 97 W ctg00898 1 3811834 -

group8 44389380 44389479 98 U 100 contig yes map

group8 44389480 45230770 99 W ctg00427 1 841291 +

group8 45230771 45230870 100 U 100 contig yes map

group8 45230871 45268999 101 W ctg00801 1 38129 -

group8 45269000 45269099 102 U 100 contig yes map

group8 45269100 45362617 103 W ctg00241 1 93518 -

group8 45362618 45362717 104 U 100 contig yes map

group8 45362718 52529442 105 W ctg00368 1 7166725 +

group8 52529443 52529542 106 U 100 contig yes map

group8 52529543 52630397 107 W ctg00849 1 100855 +

group8 52630398 52630497 108 U 100 contig yes map

group8 52630498 52731627 109 W ctg00194 1 101130 -

group8 52731628 52731727 110 U 100 contig yes map

group8 52731728 52782329 111 W ctg00297 1 50602 -

group8 52782330 52782429 112 U 100 contig yes map

group8 52782430 52995323 113 W ctg00893 1 212894 +

group8 52995324 52995423 114 U 100 contig yes map

group8 52995424 53212943 115 W ctg00632 1 217520 -

group8 53212944 53213043 116 U 100 contig yes map

group8 53213044 53252386 117 W ctg00027 1 39343 -

group8 53252387 53252486 118 U 100 contig yes map

group8 53252487 53302248 119 W ctg00824 1 49762 -

group8 53302249 53302348 120 U 100 contig yes map

group8 53302349 54880288 121 W ctg00712 1 1577940 +

group8 54880289 54880388 122 U 100 contig yes map

group8 54880389 62213084 123 W ctg00217 1 7332696 -

group8 62213085 62213184 124 U 100 contig yes map

group8 62213185 62912939 125 W ctg00045 1 699755 -

group8 62912940 62913039 126 U 100 contig yes map

group8 62913040 64709788 127 W ctg00177 1 1796749 -

group8 64709789 64709888 128 U 100 contig yes map

group8 64709889 66099063 129 W ctg00773 1 1389175 +

group8 66099064 66099163 130 U 100 contig yes map

group8 66099164 68249464 131 W ctg00531 1 2150301 +

group8 68249465 68249564 132 U 100 contig yes map

group8 68249565 70014209 133 W ctg00644 1 1764645 +

group8 70014210 70014309 134 U 100 contig yes map

group8 70014310 71270679 135 W ctg00745 1 1256370 +

group8 71270680 71270779 136 U 100 contig yes map

group8 71270780 73151104 137 W ctg00581 1 1880325 +

group8 73151105 73151204 138 U 100 contig yes map

group8 73151205 74186769 139 W ctg00995 1 1035565 +

group8 74186770 74186869 140 U 100 contig yes map

group8 74186870 74224188 141 W ctg00118 1 37319 -

group8 74224189 74224288 142 U 100 contig yes map

group8 74224289 76419705 143 W ctg00545 1 2195417 +

group8 76419706 76419805 144 U 100 contig yes map

group8 76419806 77695570 145 W ctg00804 1 1275765 -

group8 77695571 77695670 146 U 100 contig yes map

group8 77695671 77768872 147 W ctg00511 1 73202 +

group8 77768873 77768972 148 U 100 contig yes map

group8 77768973 77811828 149 W ctg00314 1 42856 +

group8 77811829 77811928 150 U 100 contig yes map

group8 77811929 77869385 151 W ctg00881 1 57457 +

group8 77869386 77869485 152 U 100 contig yes map

group8 77869486 77939272 153 W ctg00557 1 69787 +

group8 77939273 77939372 154 U 100 contig yes map

group8 77939373 78024289 155 W ctg00371 1 84917 -

group9 1 103153 1 W ctg00262 1 103153 +

group9 103154 103253 2 U 100 contig yes map

group9 103254 1535755 3 W ctg00727 1 1432502 +

group9 1535756 1535855 4 U 100 contig yes map

group9 1535856 3616491 5 W ctg00175 1 2080636 -

group9 3616492 3616591 6 U 100 contig yes map

group9 3616592 5947755 7 W ctg00258 1 2331164 -

group9 5947756 5947855 8 U 100 contig yes map

group9 5947856 9880180 9 W ctg00281 1 3932325 -

group9 9880181 9880280 10 U 100 contig yes map

group9 9880281 10045963 11 W ctg00364 1 165683 -

group9 10045964 10046063 12 U 100 contig yes map

group9 10046064 10454213 13 W ctg00927 1 408150 -

group9 10454214 10454313 14 U 100 contig yes map

group9 10454314 17102168 15 W ctg00130 1 6647855 +

group9 17102169 17102268 16 U 100 contig yes map

group9 17102269 20185685 17 W ctg00921 1 3083417 +

group9 20185686 20185785 18 U 100 contig yes map

group9 20185786 22145122 19 W ctg00251 1 1959337 -

group9 22145123 22145222 20 U 100 contig yes map

group9 22145223 22275487 21 W ctg00209 1 130265 -

group9 22275488 22275587 22 U 100 contig yes map

group9 22275588 24470562 23 W ctg00947 1 2194975 +

group9 24470563 24470662 24 U 100 contig yes map

group9 24470663 28002387 25 W ctg00465 1 3531725 +

group9 28002388 28002487 26 U 100 contig yes map

group9 28002488 28523063 27 W ctg00461 1 520576 -

group9 28523064 28523163 28 U 100 contig yes map

group9 28523164 34750879 29 W ctg00070 1 6227716 -

group9 34750880 34750979 30 U 100 contig yes map

group9 34750980 37909360 31 W ctg00793 1 3158381 -

group9 37909361 37909460 32 U 100 contig yes map

group9 37909461 37984833 33 W ctg00842 1 75373 -

group9 37984834 37984933 34 U 100 contig yes map

group9 37984934 38139203 35 W ctg00613 1 154270 +

group9 38139204 38139303 36 U 100 contig yes map

group9 38139304 38207131 37 W ctg00038 1 67828 +

group9 38207132 38207231 38 U 100 contig yes map

group9 38207232 41505823 39 W ctg00868 1 3298592 -

group9 41505824 41505923 40 U 100 contig yes map

group9 41505924 43898965 41 W ctg00815 1 2393042 +

group9 43898966 43899065 42 U 100 contig yes map

group9 43899066 44166496 43 W ctg00414 1 267431 -

group9 44166497 44166596 44 U 100 contig yes map

group9 44166597 44264445 45 W ctg00539 1 97849 +

group9 44264446 44264545 46 U 100 contig yes map

group9 44264546 44332714 47 W ctg00349 1 68169 +

group9 44332715 44332814 48 U 100 contig yes map

group9 44332815 45203941 49 W ctg00928 1 871127 -

group9 45203942 45204041 50 U 100 contig yes map

group9 45204042 45305168 51 W ctg00394 1 101127 -

group9 45305169 45305268 52 U 100 contig yes map

group9 45305269 46959593 53 W ctg00517 1 1654325 -

group9 46959594 46959693 54 U 100 contig yes map

group9 46959694 51099710 55 W ctg00146 1 4140017 -

group9 51099711 51099810 56 U 100 contig yes map

group9 51099811 51855561 57 W ctg00707 1 755751 +

group9 51855562 51855661 58 U 100 contig yes map

group9 51855662 51921363 59 W ctg01011 1 65702 +

group9 51921364 51921463 60 U 100 contig yes map

group9 51921464 53447139 61 W ctg00964 1 1525676 -

group9 53447140 53447239 62 U 100 contig yes map

group9 53447240 53470300 63 W ctg00797 1 23061 +

group9 53470301 53470400 64 U 100 contig yes map

group9 53470401 53549242 65 W ctg00151 1 78842 -

group9 53549243 53549342 66 U 100 contig yes map

group9 53549343 55724941 67 W ctg00774 1 2175599 +

group9 55724942 55725041 68 U 100 contig yes map

group9 55725042 55801688 69 W ctg00477 1 76647 -

group9 55801689 55801788 70 U 100 contig yes map

group9 55801789 55846466 71 W ctg00072 1 44678 +

group9 55846467 55846566 72 U 100 contig yes map

group9 55846567 56342941 73 W ctg00905 1 496375 +

group9 56342942 56343041 74 U 100 contig yes map

group9 56343042 56381867 75 W ctg00806 1 38826 +

group9 56381868 56381967 76 U 100 contig yes map

group9 56381968 56445894 77 W ctg00841 1 63927 +

group9 56445895 56445994 78 U 100 contig yes map

group9 56445995 56564349 79 W ctg00468 1 118355 +

group9 56564350 56564449 80 U 100 contig yes map

group9 56564450 56642819 81 W ctg00695 1 78370 +

group9 56642820 56642919 82 U 100 contig yes map

group9 56642920 56728062 83 W ctg00402 1 85143 -

group9 56728063 56728162 84 U 100 contig yes map

group9 56728163 56801901 85 W ctg00741 1 73739 -

group9 56801902 56802001 86 U 100 contig yes map

group9 56802002 57153463 87 W ctg00206 1 351462 -

group9 57153464 57153563 88 U 100 contig yes map

group9 57153564 59613103 89 W ctg00341 1 2459540 -

group9 59613104 59613203 90 U 100 contig yes map

group9 59613204 62480848 91 W ctg00784 1 2867645 +

group9 62480849 62480948 92 U 100 contig yes map

group9 62480949 63135127 93 W ctg00266 1 654179 -

group9 63135128 63135227 94 U 100 contig yes map

group9 63135228 63391389 95 W ctg00913 1 256162 -

group9 63391390 63391489 96 U 100 contig yes map

group9 63391490 63488033 97 W ctg00543 1 96544 -

group9 63488034 63488133 98 U 100 contig yes map

group9 63488134 64518953 99 W ctg00453 1 1030820 +

group9 64518954 64519053 100 U 100 contig yes map

group9 64519054 65885307 101 W ctg00397 1 1366254 -

group9 65885308 65885407 102 U 100 contig yes map

group9 65885408 68141830 103 W ctg00876 1 2256423 +

group9 68141831 68141930 104 U 100 contig yes map

group9 68141931 68223342 105 W ctg00141 1 81412 -

group9 68223343 68223442 106 U 100 contig yes map

group9 68223443 71252597 107 W ctg00720 1 3029155 +

group9 71252598 71252697 108 U 100 contig yes map

group9 71252698 71355045 109 W ctg00287 1 102348 -

group9 71355046 71355145 110 U 100 contig yes map

group9 71355146 71470442 111 W ctg00875 1 115297 -

group9 71470443 71470542 112 U 100 contig yes map

group9 71470543 71554344 113 W ctg00759 1 83802 -

group9 71554345 71554444 114 U 100 contig yes map

group9 71554445 71638064 115 W ctg00351 1 83620 +

group9 71638065 71638164 116 U 100 contig yes map

group9 71638165 71806431 117 W ctg00047 1 168267 +

group9 71806432 71806531 118 U 100 contig yes map

group9 71806532 72474149 119 W ctg00039 1 667618 +

group9 72474150 72474249 120 U 100 contig yes map

group9 72474250 72603038 121 W ctg00418 1 128789 +

group9 72603039 72603138 122 U 100 contig yes map

group9 72603139 73994077 123 W ctg00821 1 1390939 -

group9 73994078 73994177 124 U 100 contig yes map

group9 73994178 74051025 125 W ctg00950 1 56848 -

group9 74051026 74051125 126 U 100 contig yes map

group9 74051126 76750075 127 W ctg00530 1 2698950 -

group9 76750076 76750175 128 U 100 contig yes map

group9 76750176 76807781 129 W ctg00110 1 57606 +

group9 76807782 76807881 130 U 100 contig yes map

group9 76807882 76922148 131 W ctg00874 1 114267 -

group9 76922149 76922248 132 U 100 contig yes map

group9 76922249 76962113 133 W ctg00321 1 39865 +

group9 76962114 76962213 134 U 100 contig yes map

group9 76962214 77005501 135 W ctg00374 1 43288 -

group9 77005502 77005601 136 U 100 contig yes map

group9 77005602 77069284 137 W ctg00135 1 63683 +

group9 77069285 77069384 138 U 100 contig yes map

group9 77069385 77220433 139 W ctg00482 1 151049 +

group9 77220434 77220533 140 U 100 contig yes map

group9 77220534 77310380 141 W ctg00684 1 89847 -

group9 77310381 77310480 142 U 100 contig yes map

group9 77310481 77331716 143 W ctg00883 1 21236 +

group9 77331717 77331816 144 U 100 contig yes map

group9 77331817 77411466 145 W ctg00272 1 79650 +

ctg00527 1 2519 1 W ctg00527 1 2519 +

ctg00675 1 51647 1 W ctg00675 1 51647 +

ctg00124 1 62119 1 W ctg00124 1 62119 +

ctg00025 1 28389 1 W ctg00025 1 28389 +

ctg00284 1 15516 1 W ctg00284 1 15516 +

ctg00369 1 10472 1 W ctg00369 1 10472 +

ctg00936 1 27965 1 W ctg00936 1 27965 +

ctg00642 1 17904 1 W ctg00642 1 17904 +

ctg01016 1 5587 1 W ctg01016 1 5587 +

ctg00877 1 11941 1 W ctg00877 1 11941 +

ctg00946 1 14271 1 W ctg00946 1 14271 +

ctg00143 1 43246 1 W ctg00143 1 43246 +

ctg00305 1 65270 1 W ctg00305 1 65270 +

ctg00483 1 67271 1 W ctg00483 1 67271 +

ctg00102 1 30860 1 W ctg00102 1 30860 +

ctg00608 1 62318 1 W ctg00608 1 62318 +

ctg00791 1 12266 1 W ctg00791 1 12266 +

ctg00304 1 53157 1 W ctg00304 1 53157 +

ctg00812 1 70534 1 W ctg00812 1 70534 +

ctg00560 1 74004 1 W ctg00560 1 74004 +

ctg00452 1 12787 1 W ctg00452 1 12787 +

ctg00142 1 58400 1 W ctg00142 1 58400 +

ctg00809 1 2561 1 W ctg00809 1 2561 +

ctg01008 1 88229 1 W ctg01008 1 88229 +
