# Supplementary material for: High-quality reference genome of Fasciola gigantica: Insights into the genomic signatures of transposon-mediated evolution and specific parasitic adaption in tropical regions
Source: PLoS Negl Trop Dis. 2021 Oct 5;15(10):e0009750. doi: 10.1371/journal.pntd.0009750 (PMC8519440; doi:10.1371/journal.pntd.0009750)
Supplement: Attachment — Submitted filename: Response to reviewers.doc [file pntd.0009750.s027.doc]

**Dear editors and reviewers**：

Thank you for your letter and for the reviewers’ comments concerning our manuscript. It is our great honor for our manuscript to be reviewed and considered to be revised. These comments are all valuable and very helpful for improving our manuscript, as well as the important guiding significance to our further researches. We have studied all comments carefully and revised our manuscript word by word. All of the suggestions have been revised and highlighted in the manuscript. We hope that the revised manuscript will qualify enough to be accepted by the reviewers and editors. The responses to the comments are listed in the following point-by-point.

Besides, we edited the order of affiliations.

**Methods**

-Are the objectives of the study clearly articulated with a clear testable hypothesis stated?
-Is the study design appropriate to address the stated objectives?
-Is the population clearly described and appropriate for the hypothesis being tested?
-Is the sample size sufficient to ensure adequate power to address the hypothesis being tested?
-Were correct statistical analysis used to support conclusions?
-Are there concerns about ethical or regulatory requirements being met?

**Reviewer #1**:

1. How many parasites were used for the DNA extraction and from which life cycle stage? If more than one parasite was used detail how the sequencing libraries were prepared and from which samples.

A1: Thanks a lot for the reviewer’s questions. We only used one Fasciola gigantica at adult stage achieved from the buffalo liver in all the process of DNA extraction. Owing to the large size of the parasite, it is no need to consider the DNA content in this analysis.

2. Lines 454-456 - detail genome/transcriptome assemblies used here, provide accession/identifier numbers.

A2: Thanks a lot for the reviewer’s questions. We have written the genome assembly process at lines 408-421, and the transcription assembly at line 435-438 in detail. The accession number have been written at line 524-532. For the convenience of reading, we add the accession number at line 421.

Adding information: “The whole genome assembly (contig version) have been deposited in the Genome Warehouse in BIG Data Center under accession number GWHAZTT00000000 and NCBI under Bioproject PRJNA691688.”.

3. Line 502 - include details of the 8 genomes used here - species names and genome identifiers.
A3: Thanks a lot for the reviewer’s questions. We have written the information at result, while it is absent in the method section. We add the information at line 503.

Adding information: “, including F. gigantica (our assembly), F. hepatica (NCBI: GCA_002763495.2), Fasciolopsis buski (NCBI: GCA_008360955.1), Clonorchis sinensis (NCBI: GCA_003604175.1), Schistosoma mansoni (NCBI: GCF_000237925.1), Taenia multiceps (NCBI: GCA_001923025.3), swamp buffalo (BIG sub: GWHAAJZ00000000), and human (NCBI: GCF_000001405.39)”.

**Reviewer #2**: Major points:
1. Lines 469-470: The Authors should clarify which species were uses to produce single copy orthologs and where the underlying evidence to estimate the speciation events among these species was collected from? Did the author have for instance estimates from fossil data for calibration? Also, the authors should give more detailed description for the calculation of mutation rate because plain referral to MCMC and CDS alignments do not make the method repeatable for other researches.
A4: Thanks a lot for the reviewer’s questions. It is our carelessness to describe the method here. We used single copy genes among 8 species and the age of Animals (602–661 Ma) estimated in a previous fossil-calibrated eukaryotic phylogeny to estimate mutation rate. In fact, the method was nearly the same as the gene family analysis, excepting that we used global clock instead of independent rates in the MCMCtree. We add the information at line 470.

Adding information: “We used 559 single-copy orthologs multiple sequence alignment among 8 species produced in the latter gene family analysis, and estimated the mutation rate using MCMCtree with global clock. A Markov chain Monte Carlo (MCMC) process was run for 2,000,000 iterations, with sample frequency of 100 after a burn-in of 1,000 iterations. The median of simulated data was selected as mutation rate (mu = 1.73×10-9).”.

**Reviewer #3**: The idea behind the study is clear and sound.
Methods in genome sequencing, assembly and annotation seem appropriate.
Some of the downstream analysis would need a bit of improvement
A5: Thanks a lot for the reviewer’s advice.
--------------------

**Results**
-Does the analysis presented match the analysis plan?
-Are the results clearly and completely presented?
-Are the figures (Tables, Images) of sufficient quality for clarity?

**Reviewer #1**:

1. Lines 42 and in the results section - the authors describe how they identified ES proteins that were used to investigate protein-protein interactions between host and parasite.
a. How were these proteins identified/classified?

A6a: Thanks a lot for the reviewer’s questions. The method of identifying ES proteins was in the method section at line 479-493. In brief, we identified proteins with a signal peptide sequence but without a transmembrane region or the mitochondrial sequences.

Method at line 479-493:” Proteases and protease inhibitors were identified and classified into families using BLASTp (e-value < 10-4) against the MEROPS peptidase database (merops_scan.lib; (European Bioinformatics Institute (EMBL-EBI), Cambridge, UK)), with amino acids at least 80% coverage matched for database proteins. These proteases were divided into five major classes (aspartic, cysteine, metallo, serine, and threonine proteases). E/S proteins (i.e., the secretome) were predicted by the programs SignalP 5.0 [85], TargetP [86], and TMHMM [87]. Proteins with a signal peptide sequence but without a transmembrane region were identified as secretome proteins, excluding the mitochondrial sequences.”.
b. Did the authors base their predictions solely on whether the proteins contained a signal peptide and therefore would be likely to be secreted?

A6b: Thanks a lot for the reviewer’s questions. The detailed method was replied in A6a. The method was mainly predicted by bioinformatics analysis and integrated multiple databases. It identified proteins with a signal peptide sequence but without a transmembrane region or the mitochondrial sequences.
c. Analysis from F. hepatica ES proteome studies has shown that proteins that do not have signal peptides are also present in the ES products, particularly within the EVs. Did the authors check their predicted ES proteins to known ES proteome datasets for Fasciola to confirm this list?

A6c: Thanks a lot for the reviewer’s questions. We have checked our predicted ES proteins with the proteomics from *F. hepatica* extracellular vesicles by Eduardo etc. (<https://doi.org/10.1371/journal.pntd.0007087>) by mannual. There are 21 overlapping proteins relating to EV biogenesis and vesicle trafficking. We have presented the genes at line 221-224 in detail.
d. The authors should mention that these proteins represented the predicted secretome/ES proteins rather than stating these are ES proteins, unless confirmed. This data also impacts on how the PPI data should be interpreted.
A6d: Thanks a lot for the reviewer’s advice. We follow reviewer’s advice, and edit related words at result and abstract at line 43,245.

2. Figures - all the figures are composite figures - the authors should modify the font size of the text so that it will be visible upon publication.
A7: Thanks a lot for the reviewer’s advice.

**Reviewer #3**: The article provide a significant improvement in the knowledge of the genome of F.gigantica, that might be a very useful resource.
In general the results are presented in a clear way, although some clarifications and improvements can be made as detailed below.
In general the figures are of good quality, the supplementary information provided in tables need improvement, particularly by providing a reasonable annotation of individual recognizable genes.
A8: Thanks a lot for the reviewer’s advice.
--------------------

**Conclusions**
-Are the conclusions supported by the data presented?
-Are the limitations of analysis clearly described?
-Do the authors discuss how these data can be helpful to advance our understanding of the topic under study?
-Is public health relevance addressed?

**Reviewer #1**: (No Response)

**Reviewer #3**: Most of the ideas behind the article are sound and supported by the data, although some analysis might need to be revised.
The discussion in general stress relevant points, that are open for discussion
The subject is relevant and the data generated might be a valuable resource
A9: Thanks a lot for the reviewer’s advice.
--------------------

**Editorial and Data Presentation Modifications?**
Use this section for editorial suggestions as well as relatively minor modifications of existing data that would enhance clarity. If the only modifications needed are minor and/or editorial, you may wish to recommend “Minor Revision” or “Accept”.

**Reviewer #1**:

1. Line 28 and throughout the manuscript – fascioliasis should not be in italics, however, Fasciola should be in italics.

A10: Thanks a lot for the reviewer’s advice. We accepted and edited them in standard.

2. Line 59 – prevalence of F. gigantica infection.

A11: Thanks a lot for the reviewer’s advice. We accepted and edited them in standard.

3. Line 208 – sentence should read as ‘genes have’ or ‘gene has’ depending on the number of genes the authors are referring to.

A12: Thanks a lot for the reviewer’s advice. We accepted and edited them in standard.

4. Line 333 - of the rapidly evolving

A13: Thanks a lot for the reviewer’s advice. We accepted and edited them in standard.

5. Line 364 – networks

A14: Thanks a lot for the reviewer’s advice.

6. Lines 226-227 - rationale for abbreviation AAASs when the abbreviation for aminoacyl tRNA synthetases is typically AARSs- also why is the abbreviation in italics? The AARSs abbreviation is used on line 227 but is not defined.

A15: Thanks a lot for the reviewer’s advice. We accepted and edited them in standard.

7. References:
a. Lines 85-86 – Reference [1] is not the correct reference for this statement relating to the mode of action of TCBZ in disrupting beta tubulin polymerization.
b. References 7 and 45 are the same; References 10 and 29 are the same.
A16: Thanks a lot for the reviewer’s advice. We accepted and edited them in standard.

**Reviewer #2**: Minor points
1. Line 165: would -> would be

A17: Thanks a lot for the reviewer’s advice. We accepted and edited them in standard.

2. Line 281: is mainly -> mainly

A18: Thanks a lot for the reviewer’s advice. We accepted and edited them in standard.

3. Line 304: emphasize -> emphasizes

A19: Thanks a lot for the reviewer’s advice. We accepted and edited them in standard.

4. Line 469: 173x10-9 -> 173x10-9 per basepair per year
A20: Thanks a lot for the reviewer’s advice. We accepted and edited them in standard.

**Reviewer #3**: A minor suggestion, I feel that swapping some paragraphs in the intro might improve the understanding without changing the meaning.
Fisrt sentence of last paragraph (lines 92-93) would be better after line 62, that can be followed by the 3rd paragraph of the intro (lines 77 -91). This would join all the info on the biology of the parasite, before presenting the status on trematode omics and particularly Fasciolidae data (lines 63-76).
A21: Thanks a lot for the reviewer’s advice. We tried to adjust this sentence, while it would result to imcompleteness of last paragraphs. We are inclined to stay the same.

--------------------

**Summary and General Comments**
Use this section to provide overall comments, discuss strengths/weaknesses of the study, novelty, significance, general execution and scholarship. You may also include additional comments for the author, including concerns about dual publication, research ethics, or publication ethics. If requesting major revision, please articulate the new experiments that are needed.

**Reviewer #1**: The manuscript by Luo and colleagues describes a high quality genome assembly for Fasciola gigantica. This genome dataset represents an improved dataset for the tropical liver fluke, which has been assembled into 10 potential chromosomes. This study paves the way for improvements for the other Fasciola species genome allowing future comparatative analyses and provides novel insight for liver fluke biology. The manuscript is well written and is suitable for publication in PLoS NTD.
A21: Thanks a lot for the reviewer’s advice.

**Reviewer #2**: This manuscript introduces reference genome for a socio-economically important trematode Fasciola gigantica and hypothesizes both the role of both transposon expansion event and uses single copy gene to infer speciation events between Fasciola and Fasciolopsis and between Fasciola gigantica and Fasciola hepatica, respectively. Moreover, the authors hypothesize the role of gene family expansions to the size of the trematode and its development to epidemic in tropical and subtropical regions.
The assembled genome for F. gigantiga described in this manuscript clearly has major importance to the community studying parasites and the diseases they cause. The manuscript is well written, the methods for the assembly and annotation follow currently known best practices (I could only slightly criticize the use of SNAP in de novo prediction).
Apart from the required clarification to the calculation of the mutation rate, and few minor enhancements, there are not objections to publish the manuscript.
A21: Thanks a lot for the reviewer’s advice. The revise of mutation rate part was replied in A4.

Adding information: “We used 559 single-copy orthologs multiple sequence alignment among 8 species produced in the latter gene family analysis, and estimated the mutation rate using MCMCtree with global clock. A Markov chain Monte Carlo (MCMC) process was run for 2,000,000 iterations, with sample frequency of 100 after a burn-in of 1,000 iterations. The median of simulated data was selected as mutation rate (mu = 1.73×10-9).”.

**Reviewer #3**: The manuscript presented by Luo et al provides a novel assembly of the genome of the liver fluke Fasciola gigantica, and analyze the plausible role of transposons in driving adaptation in this species.
The work is well written and contributes a good wealth of data, providing an almost chromosome level assembly of this relevant species, with a good deal of info on supplementary data, that would undoubtedly move forward the knowledge of Fasciolidae biology. I feel the article has a lot of strengths, but also some weakness that might be improved a bit in order to get it published. These lay essentially in the analysis of the repeats and the prediction of interactions between parasite and host genes, and the absence of an annotated list of genes that should be provided.
Anyway I provide below a more detailed account of doubts and questions that I feel can clarify the findings and their relevance.
1. The first section describes the novel assembly that integrates long reads, paired reads and HiC data. This results in a slightly larger genome than previously published (refs 5-8), consistent with a better resolution of probably collapsed repeat sequences that here are resolved. Gene content is similar to previously described genomes of Fasciolidae, although functional annotation results in roughly 2/3 of genes with assigned function (either on Interpro, GO or KEGG). I wonder from table S7 how many genes have no associated function whatsoever, is a minor point, but would stress the size of the unknown bin.

A22: Thanks a lot for the reviewer’s question. In our analysis, we observed distinct mapping rate between different blast mapping parameters. In table S7, there are only 65.22% genes annotated by Swissprot (blastp e<10-4), while it would increase to 98.54% using default parameters. In my opinion, the main reason of existing no associated function gene is the distinct sequences similarity between Fasciolidae and core database in InterPro or the different standard in mapping. By combining multiple result, much genes could be annotated.

2. When looking at all the supplementary data, I noticed that an annotation file with the identified genes and their putative annotation is missing. This is a relevant tool that should be included, either as a supplementary table and/or as a gff annotation file with the genome assembly.

A23: Thanks a lot for the reviewer’s question. We have uploaded both genome sequences and gff annotation on BIG Sub. It can be obtained in <https://bigd.big.ac.cn/gwh/Assembly/17989/show>. The gff file contain original gene id and corresponding function annotation. We only upload genome sequence without gff file on NCBI.

3. The notion that genes were larger in Fasciolidae than in other trematodes (lines 129-131) have already been advanced, and figures S3 and s4 are almost identical to figure 1C from Choi et al, ref 7, that also includes a comparison of intron lengths. This should be properly referred and discussed. By the way ref 7 and 45 are the same…please correct.

A24: Thanks a lot for the reviewer’s advice. We add discussion at line 350.

Adding infortion: “In previous study of Fasciolidae family, Choi etc. have discovered TE expansion in Fasciola, which also explained the large lineage-specific genome size and longer annotated gene. We confirmed the result in F. gigantica genome and further identified signatures of selection based on unbalanced distribution of inserted TEs between intronic and intergenic regions in history. Especially, the strongest selection signal occurred in the speciation between the Fasciola and Fascioloides—a habitat switch from the small intestine to the liver in the host—during the PETM, which accompanied by LINE expansion biased toward intronic regions. This unexpected event provided a new evidence of adaptive evolution driven by transposition events and will prompt investigations of how such differences contribute mechanistically to the morphological phenotypes of liver flukes and related species.”.

4. The second section focus on the repeats expansions that resulted in larger genomes. The authors estimate two expansions events and time them to 12 and 65 Ma (lines 153-155). I feel that the procedures for the estimation of these times need to be further clarified.

A25: Thanks a lot for the reviewer’s advice. The method of transferring Kimura divergence in RepeatMasker to age were written at line 466-468. Indeed, there are some issues in calculating mutation time. We have followed advice from reviewer 1 and edited the method part of calculating mutation rate at line 468 (replied in A4).

5. The authors made the reasonable hypothesis that they might be underlying the increase in genome size, and probably providing evolutive advantages (lines 156-160). This idea, and the distribution of repeats between intergenic and intronic regions (next point) were also previously advanced by Choi et al, and the similarities and differences should be acknowledged.

A26: Thanks a lot for the reviewer’s advice. We add discussion at line 350. The content is the same as reply in A24.

6. The comparison of the distribution of repeats between intronic and intergenic regions (lines 161-185) is a reasonable thing to do, but I feel that the description of the procedures and results are not fully clear. I assume that “the relative proportion of TEs between intronic or intergenic regions” is calculated in all intronic vs all intergenic repeats. What is the “inferred size” of these regions? What is the corrective repeat ratio that appears in Fig 2B and how is calculated?

A27: Thanks a lot for the reviewer’s question. In my opinion, the relative proportion of TEs between intronic or intergenic regions was continuous changing accompanied with TE expansion. Therefore, we split all repeats based on inserted time into 50 time points to calculate the intronic vs intergenic ratio for different types of TE. To further correcting the result, we inferred all intronic vs all intergenic region ratio in the 50 time point as background to correct the former result. We add the method at line 466.

Adding information: “We inferred the time of LINEs insertion by transferring Kimura divergence in RepeatMasker to age (t=d/2mu). The distributions of TE elements were calculated with sliding windows (n=50). In each sliding window, we calculated the relative proportion of TE between intronic and intergenic regions, and further corrected using the whole ratio between intronic and intergenic regions.”.

7. The idea of purifying selection acting on intronic regions, except for LINEs (line 172) would imply that a statistical significant difference in TE types within intron exists in comparison with intergenic repeats. I feel this is missing, or I cannot figure out this from fig 2B.

A28: Thanks a lot for the reviewer’s question. In this manuscript, the ratio above one would be the signal of selection, and ration below one would be signal of purifying selection. Anyway, it is causing a little bit of confusion somewhere. We will replace the “purifying selection” to “purifying effect” to obtain better expression. In Fig. 2B, it is obvious to observe a peak around PETM with strong selection.

Fig. 2B

8. While this transmits the idea of LINEs being enriched in intronic sequences, a different picture has been presented previously by Choi et al, showing LINE enrichment both intronic and intergenic (see Fig.2B of ref 7) but possibly consisting of different LINE types. Are the results consistent to this? It would be good to discuss it.

A29: Thanks a lot for the reviewer’s question. Owing to the effect of purifying effect, the TE sequences should be enriched in intergenic regions. On the contrary, we observed an unusual enrichment at intronic regions in specific period, which was likely driven by selection. Fig. 2B in Choi etc. showed the overall ratio between intronic and intergenic region by current genome state. It wouldn’t reflect the dynamic of genome size in history. We inferred the ratio by calculating 50 time points in history. It is not contradictory. Types of LINEs may play an important role. However, the function of each types in parasites are also unknown, and the classification in non-model species remain deviation. Therefore, we didn’t put them in the results.

9. The idea of selecting a set of enlarged Fasciola introns and compare to other trematode species is a good one, and searching for functions through GO enrichment also is reasonable (187-197), but I feel that the functions highlighted do not reflect the diversity described in Fig 2C or in the supplementary tables (by the way, they are slightly different, there are missing categories in one or the other).

A30: Thanks a lot for the reviewer’s question. We have put many instances to show the enrichment relating to molecules internalizing substances from their external environment. We emphases genes involved in membrane transport because helminth parasites interfere with the host immune system by secreting molecules from surface tegument or gut. In GO enrichment, there are“synaptic membrane” (P = 3.52E-04), “clathrin-coated vesicle membrane” (P = 1.08E-03), and “synaptic vesicle” (P = 3.02E-03), as well as vesicles secretion processes, such as “endocytosis” (P = 7.06E-06), “Golgi organization” (P = 7.45E-05), “COPII vesicle coating” (P = 2.72E-04), “intracellular signal transduction” (P = 5.16E-04), and “endosomal transport” (P = 2.47E-03).

10. Although is a good proxy to what might be happening, I found strange that some highly represented categories as signal transduction was not highlighted, especially considering that components such as protein phosphorylation or GTPase activators are also enriched.

A31: Thanks a lot for the reviewer’s advice. We add discussion at line 203.

Adding information: “Besides, proteins relating to phosphorylation and GTPase activators were also enriched, such as "Protein phosphorylation" (p=1.73E-03), "Regulation of small GTPase mediated signal transduction" (P = 1.21E-03), "GTPase activator activity" (P = 1.13E-10).”.

11. Several questions come to my mind on the finding of particular groups of genes like tRNA synthetases (lines 225-231), or histone modificators (lines 234-240) as enriched in long introns. For example, are there are other copies of these genes in the genome that were not affected by the inclusion of long introns? Are the large introns in a particular position (more 5’ or 3’) within the genes? Intersting…

A32: Thanks a lot for the reviewer’s question. We split all genes into 10 parts, and estimated the frequency of LINEs insertions between 41 Ma and 62 Ma (>100bp in each insertion). However, we didn’t observe any enrichment in the 10 bins.

12. The fourth section attempts to find genes that interact with those of the host, and here is where I have more concerns. First proteases and inhibitors are identified by comparison with the merops database (lines 244-257), however, the annotation provided does not allow to compare if these results have, for example improved the annotation of these relevant gene families in relation to previously published assemblies (refs 5-8). By analyzing the supplementary tables I noticed that the gene annotation based on this assembly is not provided. The tables provides access to merops entries, but does not correlate with annotated genes in FGIG. I assume that repeated merops entries that appear in the table correspond to diverse genes in the FGIG genome that are best match to these merops entries, but this need to be clarified. Gene names should be corrected providing a unique identifier for each gene as well as their putative annotation. This is particularly relevant in gene families as for example those that appear in suppl table 15. It seems that approximately 17 Legumain genes have been found, but if all share the same name is impossible to evaluate for example if they are differentially expressed in diverse life stages or tissues. A complete annotation of the genes list should be provided as previously stated (in point 2).

A33: Thanks a lot for the reviewer’s question. In the supplementary section, it is common and reasonable to use annotated function gene name or merops entries, like SOD and MER0036548, instead of the original gene name, like gig1. Because it provided clear gene information for analysis. For advanced analysis of specific gene, we suggested that researchers download the annotation file in BIG Sub rather than using the gene names in the supplementary file directly (GFF file: <https://bigd.big.ac.cn/gwh/Assembly/17989/show>).

13. The major concern is however, the assumption of interactions based on the protein-protein interaction (lines 260-280). I feel this analysis is essentially incorrect. Interactions were inferred by selecting host intestinal and liver surface and secreted proteins, and those also expressed by the parasite. I wonder how many of the FGIG surface proteins mapped to human counterparts. Beside this, the interactions reported seem to be based on those described for the mammalian counterparts of the FGIG proteins. These are proved interactions within mammals, but there is no line of evidence so far that the same interactions would take place with heterologous proteins from the parasite. Extreme care should be taken into inferring biologically significant interactions without further experimental evidence. I feel this whole section is over interpreting the results.

A34: Thanks a lot for the reviewer’s advice. It is a common method to analyze the protein-protein interactions between the parasite and host. There are many researches using the same method, like Yibo Hu etc. (<https://doi.org/10.1093/molbev/msaa243>) and Jumoke Soyemi etc. (<https://doi.org/10.2174/1574893613666180108155851>). To eliminate the ambiguity, we added related reference at line 265.

14. I also have some doubts about the gene family analysis, since some of the F.gigantica specific families highlighted correspond to quite conserved GO functions, that surely are present in F.hepatica and other trematodes. Consequently they should represent duplication events exclusive of F.gigantica, that can be easily analyzed in more detail. Unfortunately the poor annotation of genes does not allow to test this since rather than having a list of genes that constitute the family we have a database hitname repeated as many times as copies. As already mentioned good annotation would allow a better understanding of the results.
A35: Thanks a lot for the reviewer’s advice. In the analysis of gene family, we used all proteins from *F.hepatica*, and identified specific duplication in *F.gigantica*. The phylogenetic tree also proved specific gene duplication. Although we couldn’t exclude all errors by constructing phylogenetic tree for all families, it is reasonable to believe the result. As the same as reply in A33, we suggested that researchers download the annotation file in BIG Sub rather than using the gene names in the supplementary file directly.

In general I feel that the article has interesting novel data, but have some weakness in the analysis, most of them that can be reasonably sorted out before publication.

--------------------

PLOS authors have the option to publish the peer review history of their article ([what does this mean?](https://journals.plos.org/plosntds/s/editorial-and-peer-review-process#loc-peer-review-history)). If published, this will include your full peer review and any attached files.
Yes.


If you choose “no”, your identity will remain anonymous but your review may still be made public.


**Do you want your identity to be public for this peer review?** For information about this choice, including consent withdrawal, please see our [Privacy Policy](https://www.plos.org/privacy-policy).

Reviewer #1: No

Reviewer #2: No

Reviewer #3: No


Figure Files:
While revising your submission, please upload your figure files to the Preflight Analysis and Conversion Engine (PACE) digital diagnostic tool, https://pacev2.apexcovantage.com. PACE helps ensure that figures meet PLOS requirements. To use PACE, you must first register as a user. Then, login and navigate to the UPLOAD tab, where you will find detailed instructions on how to use the tool. If you encounter any issues or have any questions when using PACE, please email us at figures@plos.org.
 
Data Requirements:
Please note that, as a condition of publication, PLOS' data policy requires that you make available all data used to draw the conclusions outlined in your manuscript. Data must be deposited in an appropriate repository, included within the body of the manuscript, or uploaded as supporting information. This includes all numerical values that were used to generate graphs, histograms etc.. For an example see here: http://www.plosbiology.org/article/info%3Adoi%2F10.1371%2Fjournal.pbio.1001908#s5.
 
Reproducibility:
To enhance the reproducibility of your results, we recommend that you deposit your laboratory protocols in protocols.io, where a protocol can be assigned its own identifier (DOI) such that it can be cited independently in the future. Additionally, PLOS ONE offers an option to publish peer-reviewed clinical study protocols. Read more information on sharing protocols at https://plos.org/protocols?utm_medium=editorial-email&utm_source=authorletters&utm_campaign=protocols

__________________________________________________
In compliance with data protection regulations, you may request that we remove your personal registration details at any time. (Use the following URL: https://www.editorialmanager.com/pntd/login.asp?a=r). Please contact the publication office if you have any questions.
