# Supplementary material for: High-quality reference genome of Fasciola gigantica: Insights into the genomic signatures of transposon-mediated evolution and specific parasitic adaption in tropical regions
Source: PLoS Negl Trop Dis. 2021 Oct 5;15(10):e0009750. doi: 10.1371/journal.pntd.0009750 (PMC8519440; doi:10.1371/journal.pntd.0009750)
Supplement: Attachment — Submitted filename: 2nd-Response to reviewers.doc [file pntd.0009750.s028.doc]

**Dear editors and reviewers**：

Thank you for your letter and for the reviewers’ comments concerning our manuscript. It is our great honor for our manuscript to be reviewed and considered for publication. Questions in the list would be responded and annotation issues would be fixed by adding both gene id and function name. All of the suggestions have been revised and highlighted in the manuscript. We hope that the revised manuscript will qualify enough to be accepted by the reviewers and editors. The responses to the comments are listed in the following point-by-point.

**Reviewer #1**: The authors have addressed the comments raised by the previous review - however several of the points included in their response have not been incorporated into the manuscript (see below). Following the edits/comments below the manuscript will be suitable for publication.

1. For example - the authors stated that only one adult fluke was just for the sequencing. This needs to be included in the methods. Please include where relevant the points raised in the review in the text.

A1: Thanks a lot for the reviewer’s suggestion. The certain number of sequencing sample was added at LINE 431.

Adding information: “For the reference genome sequencing, one F. gigantica at adult stage was derived from infected buffalo in the Guangxi Zhuang Autonomous Region.”.

2. Also it was difficult to see where the changes were made as the authors used the comment box on the pdf to add in their changes. The edits apparently made in the highlighted manuscript, such as lines 161-172 relating to the purifying selection, were not incorporated in the final version. In the next review please include a highlighted manuscript using tracked changes or highlighting the text in colour so that the changes can be followed.

A2: Thanks a lot for the reviewer’s questions. It is our carelessness. We incorporated them in the final version, and highlighted these words in yellow at highlighted version.

3. Line 105 - The reference included for the beta tubulin polymerization is still not right. Include a relevant reference.
A3: Thanks a lot for the reviewer’s suggestion. We changed the reference, and used the article “Understanding triclabendazole resistance.” (doi: https://doi.org/10.1016/j.yexmp.2007.01.009).

4. Lines 221-224 - Although the authors state that they checked their predicted secreted sequences against the EV proteins published by de la Torre et al., the authors should also check their data against the available secretome data not just the EV proteins, to identify the actual proteins present in the ES products.

A4: Thanks a lot for the reviewer’s suggestion. At lines 221-224, we firstly found that genes with LINE insertions were enriched with membrane-associated proteins, especially relating EV biogenesis. It is the reason that we only compared our result with EV proteins published by de la Torre et al. rather than whole secretome data. However, it is a good suggestion to validate the subsequent result in E/S protein identification. We additionally downloaded secretome data generated by the mass spectrometer from Lucía Sánchez Di Maggio et al. (“Across intra-mammalian stages of the liver f luke Fasciola hepatica: a proteomic study”, ProteomeXchange ID: PXD004418). We aligned 689 *F. hepatica* proteins identified in the article to 852 predicted E/S proteins in my study using blastp with default parameter. 679 *F. hepatica* proteins can be found in my E/S database, and 425 *F. hepatica* proteins had E-value less than 1, which indicated fair mapping quality. We added the result at Supplementary material-Figures S8.

5. Line 309-310 - how did the authors perform this analysis? Is this based on their predicted secretome data or from proteomic analysis?

A5: Thanks a lot for the reviewer’s question. It is based on the predicted E/S proteins identified by bioinformatics method. There are many genes belonging to CPY450 family in the gene annotation. However, we didn’t find such genes in the predicted E/S proteins in *F. gigantica.*

6. Line 383 - amend the sentence to: Choi et al.

A6: Thanks a lot for the reviewer’s suggestion.

7. Lines 544-547 - The species names in this section should be in italics.

A7: Thanks a lot for the reviewer’s suggestion.

**Reviewer #3**: COMMENTS ON REVISED VERSION OF LUO ET AL.

While most of the issues and questions have been considered, some of the main issues are still there, and need to be corrected in order to make the manuscript acceptable for publication.

8. The main issue is still the poor annotation, devoid of unique Identifiers for the genes, that make most of the data presented in tables and figures completely unfollowable. While the manuscript is an excellent effort assembling a chromosome level genome, the usefulness of this resource for the community upon publication would be limited by this issue, making it not comparable to other assemblies available.

Following one of the suggestions a gff table is included (A23). While that provides a detailed information for those more interested, is not a solution for the simple reader interested in looking the information of any one of the genes that appear in tables 9-16. There is no place to go, and since genes are named based on their hits, when these are repeated (as frequently occur) there is no way of identifying a particular gene and differentiate it from other members of the same family. Is impossible to pretend that each reader interested in a particular gene should download and filter a gff table (as suggested in responses A33 and A35). Providing appropriate and clear information is a task of the authors, not the readers.
A8: Thanks a lot for the reviewer’s suggestion and criticism. We added both gene id and the corresponding function id in the supplementary table, for example “gig1584_mRNA1(SOD1);”. We also added Table S17 in supplementary file to present 8020 genes in *F. gigantica* annotated by human uniprot dataset using blastp with E-value<10^-4^.

.

9. A very simple solution for this is a table with the 12503 identified genes each with a unique IDs and a definition. Beside this, all tables should include gene IDs (it can also combine names but unique identifiers are mandatory) in order to make it useful and verifiable. This is a main issue and should be solved before publication. The work has reached excellent results assembling the genome, and annotation, and it would be a pity if published in a way that requires other researchers to run complex filtering steps and/or reanalize the data to find information that the authors already have, and should be made available in a way that could be easily used by the community.

A9: Thanks a lot for the reviewer’s suggestion and criticism. The content is the same as reply in A8.

10.	The annotation weakness also compromises the idea of the protein-protein interaction analysis. I agree as stated in response A34 that this could be an approach to gain information based on better analyzed protein interactions in host, but we have to be very careful on the design of the assay and on the interpretation of the results. In order to make this kind of analysis, we have to be sure that the proteins considered are true orthologs, ie. that the actual parasite gene considered is the unique orthologue of the host one detected in the interaction. Several of the proteins included might be part of gene families, so the question of finding the correct orthologue (and not paralogues) is not trivial. While is stated that the reciprocal blast best-hits were considered, the absence of proper identifiers make this untestable. Here again, having good unique gene ids is essential.

A10: Thanks a lot for the reviewer’s suggestion and criticism. The content is the same as reply in A8.

11. Similarly in relation to the gene family analysis, is not a question of if is reasonable to believe in the results as stated in answer 33 but rather if the information provided sustain the results. I do believe that the authors have performed well the experiments and have sound results, but I cannot find useful information in what is published, since I cannot identify different genes of the same family. Questions related to these families, their origins and similarities with those from other species remain obscure if we cannot pick the appropriate genes to compare and advance in their knowledge.

A11: Thanks a lot for the reviewer’s suggestion and criticism.

12. Other issues related to the repeats and the acknowledge and discussion of previous works in the same line have been taken into account, making minor modification in the discussion (responses A24, A26 and A29). Since some of the results here mirror those already published I would have expected them to be commented earlier than in discussion, but this is a matter of opinion.

A12: Thanks a lot for the reviewer’s suggestion. We added the result and reference by Choi at result at line 179.

13. Other questions related to repeat expansion analysis and selection (A25-28), were answered or clarified appropriately. Thanks for that.

A13: Thanks a lot for the reviewer’s advice.
